# Muscle Function and Kinematics during Submaximal Equine Jumping: What Can Objective Outcomes Tell Us about Athletic Performance Indicators?

**DOI:** 10.3390/ani11020414

**Published:** 2021-02-05

**Authors:** Lindsay St. George, Hilary M. Clayton, Jonathan Sinclair, James Richards, Serge H. Roy, Sarah Jane Hobbs

**Affiliations:** 1 School of Sport and Health Sciences, University of Central Lancashire, Preston PR1 2HE, UK; JKSinclair@uclan.ac.uk (J.S.); JRichards@uclan.ac.uk (J.R.); SJHobbs1@uclan.ac.uk (S.J.H.); 2 Sport Horse Science, Mason, MI 48824, USA; claytonh@msu.edu; 3 Delsys/Altec Inc., Natick, MA 01760, USA; sroy@delsys.com

**Keywords:** show jumping, surface electromyography, equine athlete, horse, biomechanics, jumping technique, jump training, performance analysis, equestrian

## Abstract

**Simple Summary:**

Jumping represents the most popular equestrian discipline. However, traditional selection and training strategies for jumping horses have not been validated using instrumented performance analyses to scientifically inform the optimization of athlete selection, training and competitive performance. We aimed to quantify the role of muscle function during the equine jump, its relationship to athletic performance indicators, and how this objective information can inform equestrian selection and training practices. We used three-dimensional kinematic and surface electromyography data to quantify movement and muscle activation, respectively, from horses executing a submaximal jump. Horses were grouped based on their ability to raise the center of mass during the jump suspension—a fundamental, objective measure of jumping performance. Kinematic data were used to objectively measure equestrian-derived preferences for movement traits related to impulsion, engagement and joint articulation. Horses that raised the center of mass highest during jumping displayed muscle activation and movement strategies that indicate a greater ability to rapidly generate hindlimb muscular force during jump take-off. These findings provide objective support for equestrian preferences related to the generation of engagement, impulsion and hindlimb muscle power when selecting and training jumping horses and justify their prioritization as objective performance indicators for the sport of equine jumping.

**Abstract:**

Selection and training practices for jumping horses have not yet been validated using objective performance analyses. This study aimed to quantify the differences and relationships between movement and muscle activation strategies in horses with varying jump technique to identify objective jumping performance indicators. Surface electromyography (sEMG) and three-dimensional kinematic data were collected from horses executing a submaximal jump. Kinematic variables were calculated based on equestrian-derived performance indicators relating to impulsion, engagement and joint articulation. Horses were grouped using an objective performance indicator—center of mass (CM) elevation during jump suspension (Z_CM_). Between-group differences in kinematic variables and muscle activation timings, calculated from sEMG data, were analyzed using one-way ANOVA. Statistical parametric mapping (SPM) evaluated between-group differences in time and amplitude-normalized sEMG waveforms. Relationships between movement and muscle activation were evaluated using Pearson correlation coefficients. Horses with the greatest Z_CM_ displayed significantly (*p* < 0.05) shorter gluteal contractions at take-off, which were significantly correlated (*p* < 0.05) with a faster approach and more rapid hindlimb shortening and CM vertical displacement and velocity, as well as shorter hindlimb stance duration at take-off. Findings provide objective support for prioritizing equestrian-derived performance indicators related to the generation of engagement, impulsion and hindlimb muscle power when selecting or training jumping horses.

## 1. Introduction

Show jumping is one of three Olympic equestrian sports and represents the most popular equestrian discipline, with the highest number of registered equine and human athletes and competitions within the Fédération Equestre Internationale [1,2,3]. Show jumping places great physical demands on the equine athlete; at the highest levels, horses jump 10–13 obstacles up to 1.70 m high and 2.00 m wide, and sometimes competing against the clock [4]. Thus, several years of technical training and physiological conditioning are required for a jumping horse to reach its full potential, which equates to significant investments of money, resources and time [5,6,7,8,9]. Despite this, the selection of individuals and training strategies for the equine jumping athlete are largely based on traditional, anecdotal methods [10,11]. Researchers have therefore advocated the advancement of performance analysis in equestrian sport, particularly for scientifically informing the optimization of athlete selection, training and ultimately competitive performance [12]. To accomplish this for show jumping, the development of objective performance indicators that aim to define successful performance or outcomes [13] is required [12]. Predictive indicators of future performance are particularly relevant for the equine industry, as early selection and/or training methods of jumping horses may facilitate improved competitive performance, career longevity and welfare, as well as reductions in time and financial investments required to produce successful athletes [14].

In the scientific literature, kinematic and kinetic analysis of the equine jumping effort are well described in horses with differing levels of experience/training when jumping a variety of obstacles [15,16,17,18,19,20,21,22,23]. In an effort to quantify objective performance indicators for equine jumping athletes, comparative studies have reported kinematic differences between horses judged as good or poor jumpers [16,24] and correlations between kinematic traits and experience/competition level or competition results [5,19,21,25]. To determine whether performance indicators could be detected at a young age, a longitudinal study explored the consistency of jump technique from 6 months to 5 years of age [6,7,14,26]. At 5 years old, horses were grouped by observation into “best” and “worst” jumpers, based on the ability to clear a 1.50 m fence in a ridden puissance (high jump) competition. When ridden over a 1.15 m fence, the 5-year-old best jumpers exhibited lower vertical displacement of the center of mass (CM) during the jump suspension, with greater forelimb shortening and hindlimb retroflexion to aid fence clearance, compared with the worst jumpers [6]. In another study, Powers and Harrison [16] studied untrained horses (3–5 years old), jumping unridden over a 1.0 m high and 0.5 m wide spread fence. They were judged as “good” or “poor” jumpers, based on their ability to successfully clear the fence. In contrast to Bobbert et al. [6], good jumpers exhibited significantly greater vertical displacement of CM during jump suspension than their poor jumping counterparts [16].

Interpretation of previous studies must consider methodological differences in fence type/height, ridden vs. unridden jump execution, and particularly the age and training level of the horses studied, as research has verified that jump performance traits can be altered through responses to training [7]. These alterations include improved ability to control the height and trajectory of the CM [7], suggesting that lower vertical displacement of the CM, observed in the best jumpers [6], may be a trained response. This is unsurprising, as the generation of muscular force (strength) and neuromuscular control (motor skill) ultimately represent the limiting factors for how high the CM can be raised and the horse’s ability to rotate the body around the CM, which represent the main determinants of jumping capacity/success [7,27]. However, limited information exists regarding muscle function during the equine jump, its relationship to athletic performance and kinematic indicators, and how this information can be practically applied to selection and training practices.

Surface electromyography (sEMG) provides equine biomechanists with a non-invasive tool for quantifying superficial muscle activity. sEMG has been used to study equine muscle function during normal locomotion at walk, trot and canter [28,29,30,31,32,33,34,35], but only two known studies have evaluated muscle activity during jumping [36,37]. Unfortunately, a comparison of findings between these studies is confounded by methodological variation for sEMG signal detection and processing and differences in the jumping tasks studied. Further, guidelines on best practice for equine sEMG signal processing only appeared after these publications [32,38] and were therefore not employed by either of the studies. Kinematic data were also not collected, which is recommended for developing a comprehensive understanding of the role of equine musculature during jumping [39]. These methodological limitations necessitate further sEMG research, which employs best practice for sEMG signal detection and processing and an analysis of kinematic data to provide a comprehensive quantification of equine muscle function during jumping.

To bridge the gap between science and practice, researchers must not overlook equestrian knowledge [40], which can serve as the basis for developing objective and practically relevant performance indicators for equestrian sport [12,41]. Unfortunately, this equestrian-derived information is not widely available and has not been incorporated in biomechanical studies examining equine jumping technique. In recognition of this, St. George et al. [11] used an original questionnaire to identify performance indicators and training methods employed by equestrians for show jumping horses. However, the suitability of these equestrian-derived performance indicators has not been validated using objective measures and will therefore form the basis for the kinematic outcome measures employed in this study. This will facilitate a mixed-methods approach that also includes sEMG measures of muscle function to provide further insight to potential underlying mechanisms that might influence athletic performance.

## 2. Materials and Methods

The methods incorporated both kinematic and sEMG measurements in order to fulfill the aim of quantifying the differences and relationships between movement and muscle activation strategies in horses with varying jump techniques, and to use this information to identify objective performance indicators for equine jumping. We focused the kinematic analysis on movement traits deemed important to equestrians [11] using a mixed-methods approach. To investigate differences in movement traits and muscle activation, horses were grouped based on CM elevation during the jump suspension, a commonly used objective discriminative performance indicator [27]. Finally, relationships between movement traits and both CM elevation and muscle activation were investigated using correlation analysis to determine potential underlying mechanisms that might influence jumping performance. It is hypothesized that kinematic and sEMG outcome measures will differ significantly according to elevation of the CM, which will provide objective support for some equestrian-derived performance indicators when selecting jumping athletes. A secondary hypothesis is that some kinematic and muscle activity outcome measures will be significantly correlated, which will provide objective insight on how muscle function relates to specific equestrian-derived performance indicators and overall jumping performance.

### 2.1. Horses

Seventeen horses (*n* = 17, age: 9.8 ± 2.3 years, height: 158.4 ± 8.0 cm, breed: various, sex: 7 mares, 10 geldings) with different levels of jumping skills and competition experience were employed for this study. Six (*n* = 6) horses had competed at a minimum level of British Showjumping Foxhunter up to 1.60 m international show jumping classes and were ridden by three professional riders. Six (*n* = 6) horses had lower level competition experience at jump heights ranging from 0.8 to 1.0 m and were ridden by their normal rider, each with similar experience (14–20 years riding experience, competed minimum British Eventing 80). Five (*n* = 5) horses were used in a riding school for novice to advanced riding lessons and were ridden by one experienced rider (14 years’ experience, competed at 1.0 m unaffiliated show jumping). All horses were in work at the time of data collection, were physically fit and could execute a 1.0 m fence.

### 2.2. Instrumentation and Equipment Set Up

Prior to instrumentation, horses completed a warm up consisting of walk, trot and canter. The duration was approximately 15 min but was dependent on each horse’s specific needs, as determined by the rider.

**Figure 1:**
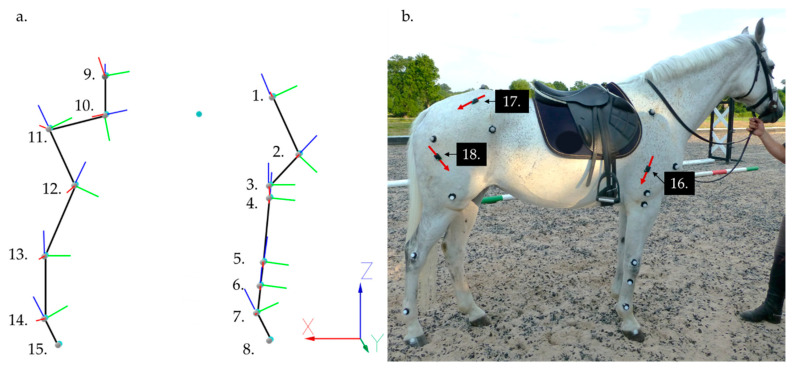
(**a**) The rigid segment model created for the subject in (**b**), showing marker locations (white spheres) and virtual landmarks (turquoise spheres), including the virtual CM maker. Black lines indicate segments. Red, green and blue lines indicate the mediolateral, anterior–posterior and dorsoventral axes, respectively, of each segment coordinate systems (SCS). Anatomical locations for marker placement are as follows: 1. proximal end of the spine of the scapula, 2. greater tubercle of the humerus (representing center of rotation of the shoulder joint), 3. lateral epicondyle of the humerus (center of rotation of the elbow joint), 4. lateral tuberosity of the radius, 5. lateral styloid process of the radius, 6. proximal end of metacarpal IV, 7. the metacarpal epicondyle (center of rotation of the metacarpophalangeal joint (MCPJ)), 8. lateral hoof wall (approximately over center of rotation of the distal interphalangeal joint (DIPJ)), 9. between the tubera sacrale, 10. most ventral part of the tuber coxae, 11. greater trochanter (center of rotation of the hip joint), 12. lateral epicondyle of the femur (center of rotation of the stifle joint), 13. talus (center of rotation of the tarsal joint), 14. the metatarsal epicondyle (center of rotation of the metatarsophalangeal joint (MTPJ)), and 15. the lateral hoof wall. (**b**) Anatomical locations for sEMG sensor placement are as follows: 16. triceps brachii, 17. middle gluteal, and 18. biceps femoris. Red arrows indicate the orientation of sEMG sensors, which ensured that electrode bars were orientated perpendicular to underlying muscle fiber direction.

Kinematic: Spherical retro reflective markers (25 mm diameter) were positioned over predetermined anatomical landmarks on the right side of each horse and are illustrated and described in Figure 1. These kinematic markers were attached using double-sided tape, after trimming excessive hair to ensure optimal adhesion. Eight infrared Qualisys Oqus cameras (Qualisys AB, Göteborg, Sweden) were positioned side by side in a linear configuration to collect three-dimensional (3D) kinematic data from the markers on the right side of the horse during multiple, consecutive strides. An extended calibration technique was conducted to produce a calibration volume approximately 8 m in length.

Electromyographic: sEMG sensors were positioned to record from the long head of triceps brachii (triceps), middle gluteal (gluteal), and vertebral head of biceps femoris (biceps femoris) muscles using wireless sEMG sensors (Trigno, Delsys Inc., Natick, MA, USA) that have a bipolar parallel bar electrode configuration and an interelectrode distance of 10 mm. Muscles were selected based on their superficial location, size and documented contribution to movement during jumping [36,42]. Prior to data collection, all hair was removed from electrode locations. Sensor sites are illustrated in Figure 1b, triceps: midway along and approximately 5 cm cranial to a line joining the olecranon and proximal point of scapular spine [43]; biceps femoris: approximately over the third trochanter and approximately 9 cm cephalad to the cranial margin of semitendinosus [44]; gluteal: approximately midway between the lumbosacral joint and greater trochanter [45]. Following warm up, sensor sites were cleaned thoroughly with isopropyl alcohol wipes. A small amount of saline solution was applied to the electrodes bars to act as an electrolytic solution [46,47]. Sensors were then positioned on the muscle belly, with the electrodes oriented perpendicular to the underlying muscle fiber direction [48,49] and attached to the skin using a combination of Delsys Adhesive Surface Interface strips (Delsys Inc., USA) and strips of double-sided tape, applied to the top and bottom of the sensor, next to each electrode pair.

### 2.3. Data Acquisition Protocol

Three-dimensional kinematic (232 Hz) and sEMG (2088 Hz) data were collected unilaterally from the right side of each horse during ridden static, canter and jump trials performed in that order. Data from static (standing) and canter trials were collected from each horse in order to create the rigid segment model (Section 2.4) and to normalize sEMG signals (Section 2.5), respectively. Data were collected from one static trial and a minimum of six canter trials for each horse, with canter three trials being collected from the left and right canter leads in random order. Horses were permitted to travel at their preferred velocity for canter trials. Jumping trial protocol is described separately below. Data were collected using Qualisys Track Manager (QTM) software (version 2018.1, Qualisys AB, Göteborg, Sweden), with sEMG and kinematic data collected synchronously using an external trigger system (Delsys Trigger Module, Delsys Inc., USA).

#### Jumping Trial Protocol

After the canter trials, a jump combination was set up within the calibrated volume. Jump set up was informed by questionnaire results from St. George et al. [11], which revealed a preference for evaluating movement in the ridden horse over a “grid line”, which is a series of fences. Two fences, a cross rail and a 1.0 m high vertical fence were therefore set approximately 11 m (two strides) apart. Kinematic and sEMG data were collected from the 1.0 m vertical fence (fence 2), which was positioned approximately 4.5 m from the cameras in the center of the calibration volume, with the cross rail set slightly before camera one to allow for the two-stride distance. This set up was employed to conform with equestrian preference and to standardize the approach to fence 2. The 1.0 m fence height was chosen because it could be executed by all horses, and the forces required for horses to jump a fence lower than this height are not much greater than those observed during canter [17,50].

Horses were permitted to warm up over lower fences (0.7–0.9 cm) prior to data collection. A minimum of six jump trials were collected from each horse, of which three trials were collected from left and right canter leads performed in random order during the jump approach. A jump trial was successful when the horse maintained the correct canter approach lead and executed the 1.0 m fence without hitting or knocking the pole. Horses were permitted to travel at their preferred velocity for the jump approach.

### 2.4. Kinematic Data Processing and Analysis

Kinematic data were tracked in Qualisys Track Manager (version 2018.1, Qualisys AB, Göteborg, Sweden) and imported into Visual3D (version 2020.07.4, C-Motion Inc., Germantown, MD, USA) software for further analysis. Kinematic data from jump trials were interpolated (maximum gap: 10 frames) and filtered using a Butterworth 4th order filter, with a 12 Hz cut-off frequency, which was determined using residual analysis. For each horse, a rigid-body model of the forelimb (FL) and hindlimb (HL) was created (Figure 1a). Virtual markers were created 2 cm medial to the tuber spinae scapulae, the ventral tuber coxae and the markers over the centers of rotation of the joints of the limbs, as described by Hobbs et al. [51]. The rigid segments were defined using anatomical and virtual marker coordinates from the static trial. A reference point on the trunk which coincides with the CM was created using a modified version of the method described by Bogert [15], where a virtual marker was projected midway between the tuber sacrale and greater tubercle of humerus markers. Rigid-body segment models were applied to all dynamic trials from the same subject. Joint angles were calculated based on the static trial using the cardan sequence x, y, z and measured in the sagittal plane, where flexion/extension was defined as rotation around the segment coordinate system (SCS) x-axis, and the flexor side defined as palmar for shoulder, carpal and stifle joints and as cranial for elbow, hip and tarsal joints.

Forelimb and hindlimb hoof impact and lift-off events were calculated from kinematic data using sagittal plane angles in accordance with Holt et al. [52]. The footfall pattern of canter is as follows: (1) trailing hindlimb (TrH); (2) leading hindlimb (LdH) and trailing forelimb (TrF) (as a diagonal pair); and (3) leading forelimb (LdF). Thus, as unilateral sEMG and kinematic data were collected from the right side, the right forelimb and hindlimb functioned as LdF and LdH during right lead canter and as TrF and TrH during left lead canter. Canter and jump strides were denoted by successive right hindlimb impact events, regardless of whether the hindlimb acted as LdH or TrH. In accordance with standardized terminology for equine jump kinematics [53], the jumping effort was denoted as approach stride 1 (A1 stride), jump and departure stride. In this study, only data from the A1 stride and jump stride were available. Kinematic gait events from the forelimb and hindlimb were applied to sEMG and kinematic signals from canter and jump trials for stride segmentation and analysis.

**Figure 2:**
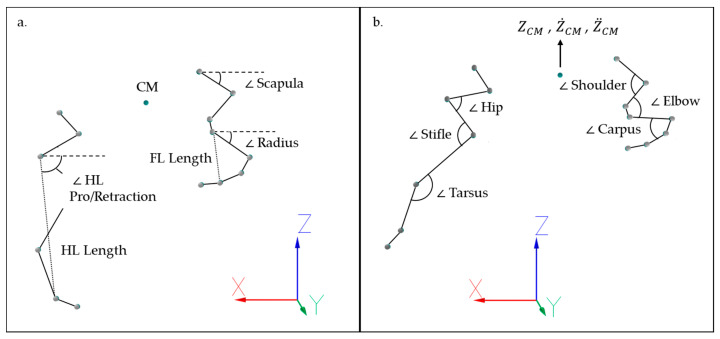
Illustration of joint angles, segment angles, segment lengths and the center of mass (CM) target used to calculate discrete kinematic variables, and the x, y, z axes of the lab coordinate system (LCS). For illustrative purposes, variables are depicted during (**a**) jump take-off and (**b**) jump suspension but were calculated across both A1 and jump strides. (**a**). scapula segment angle, radius segment angle, CM target, forelimb (FL) segment length, hindlimb (HL) segment length, pro/retraction angle of hindlimb (HL) segment (**b**) CM vertical displacement ($Z_{CM}$), CM vertical velocity (${\dot{Z}}_{CM}$), CM vertical acceleration (${\ddot{Z}}_{CM}$) and an illustration of the flexor side of shoulder, elbow, carpus, hip, stifle and tarsal angles.

Findings from St. George et al. [11] were reviewed and equestrian-derived performance indicators that could be translated into kinematic outcome measures, using available kinematic data from forelimb and hindlimb segments, were selected for this study. In accordance with St. George et al. [11], the selected performance indicators were organized under three broad themes: joint articulation, impulsion and engagement. Engagement encompassed movement traits related to increased flexion of the hindlimb joints during stance and increased hindlimb protraction, with impulsion encompassing traits related to the release of energy stored during engagement [54]. Joint articulation encompassed functional or aesthetic joint or segment movements during jump stride. Equestrian-derived performance indicators, their associated kinematic outcome measures, and calculation techniques employed in this study are summarized in Supplementary Table S1. Discrete spatiotemporal variables were calculated for A1 and jump stride and included: forelimb and hindlimb stance duration, stride duration, stride velocity and duty factor [55]. Time-series data from joint angles, segment angles, segment lengths, and vertical displacement of the CM marker (Figure 2) were used to calculate the remaining kinematic variables (Supplementary Table S1). To correct for interindividual variation between horses, joint angle data were normalized to those recorded during the static trial for each horse and are thus presented as angular changes from the standing position [56,57]. Segment angles, and linear and temporal kinematic data were not normalized. Where possible, the timings of peak joint/segment angular variables were calculated and normalized to the associated stride duration (A1 or jump stride, as specified in Supplementary Table S1).

### 2.5. sEMG Data Processing and Analysis

Raw sEMG signals were differentially amplified by a factor gain of 909, a common-mode rejection ration of >80 dB and an internal Butterworth high-pass (20 ± 5 Hz cut-off, >40 dB/dec) and low-pass filter (450 ± 50 Hz cut-off, > 80 dB/dec). Post-processing and analysis of sEMG signals was conducted in Visual3D version 2020.07.4. A constant delay of 20 ms between kinematic and sEMG data was corrected for by shifting sEMG signals forward by 5 frames prior to further post-processing and analysis. sEMG signals from canter and jump trials were direct current (DC)-offset removed and high-pass filtered using a 4th order Butterworth filter with a 40 Hz cut-off frequency [38] and full wave rectified. The quality of each sEMG signal was visually scrutinized by two researchers (L.S.G., J.R.), prior to further signal processing and analysis. Signals were excluded from the dataset where visual signs of compromised sensor adhesion or inconsistent skin contact, due to the dynamic nature of the task, were apparent through high levels of baseline and movement artefact noise contamination, which were not attenuated by the appropriate high-pass filtering techniques applied [38]. Discrete sEMG variables included the timings of sEMG peak amplitude, activity onset, offset and the resultant activity duration for each muscle. Continuous variables were in the form of amplitude and time-normalized sEMG signals during A1 and jump stride, which were analyzed using statistical parametric mapping (SPM).

**Figure 3:**
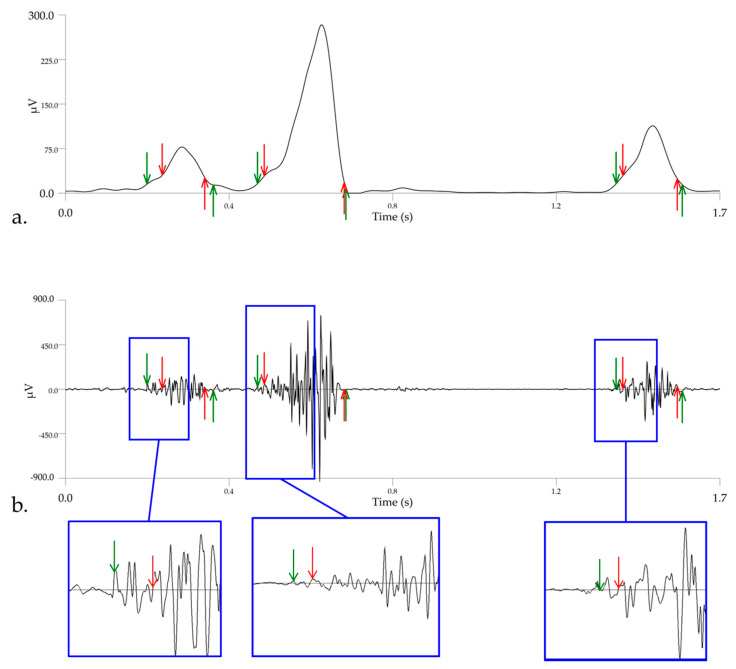
A comparative example of the improved accuracy of biceps femoris activity onset (downward arrows) and offset (upward arrows) events when a 5% amplitude threshold (green arrows) is employed compared to the 10% amplitude threshold (red arrows). (**a**) shows the enveloped sEMG signal (Butterworth 4th order low-pass filter, 10 Hz cut-off) employed for event detection, with (**b**) showing the same events applied to high-pass filtered data (40 Hz cut-off) to illustrate the accuracy of events.

Muscle activity onset and offset events were detected using enveloped signals, smoothed using a Butterworth 4th order low-pass filter with a 10 Hz cut-off frequency, to provide a clearer representation of the time-varying sEMG amplitude. This cut-off frequency was employed specifically to detect onset/offset events because we have found that it results in accurate and consistent event detection, which corresponds to activity patterns in high-pass filtered, unenveloped signals (Figure 3). A modified version of the double threshold method, described by Bonato et al. [58] and Merlo et al. [59], was employed for detecting onset and offset events. This method involves the application of timing and amplitude detection thresholds to conditioned signals, which allows the user to establish probabilities for detecting false positives and actual events. In this study, the amplitude threshold was defined as 10% of the peak amplitude value of each individual sEMG signal [43,60] and the timing threshold was defined as 5% of the average gait cycle duration [61] across all horses. In the initial detection process, muscle activity onset and offset events were detected as the point where the signal exceeded or was less than 10% of the peak amplitude threshold, respectively, for a time duration greater than 5% of the gait cycle. For example, the muscle was considered “inactive” when the onset deviated from the amplitude threshold for less than 5% of the gait cycle and was rejected as a false positive. In the post-processing procedure, temporal distances between offset and onset events, which were less than 5% of the gait cycle were considered to belong to the same contraction and removed as false positives. The 5% timing threshold was calculated and applied separately for A1 and jump strides to reflect the known significant differences in stride duration [62]. Further, the 10% amplitude threshold was reduced to 5% to improve accuracy for certain horse/muscle combinations where lower baseline activity was observed and an example of this is provided in Figure 3. Following post-processing, data were visually checked to ensure that marked artefacts were not falsely detected as onset events in accordance with previous human studies [63]. Onset and offset events and resultant activity duration for each muscle were normalized to the respective percentage of A1 or jump stride. Timing of peak amplitude for each stride was detected from the enveloped (10 Hz) signals and normalized to percent stride.

The maximal signal observed during canter was employed as the reference voluntary contraction (RVC) for normalizing sEMG signals. This RVC was selected because the horse normally approaches the jump in canter [53], so it permitted examination of the proportional change in muscle activity between canter and the jumping effort. The peak sEMG amplitude of each canter stride was calculated from enveloped signals (Butterworth, 4th order filter, 25 Hz cut-off frequency) from each muscle. Then, the maximum canter amplitude value within each horse, muscle and limb (LdH, TrH) were used to normalize corresponding sEMG data from jump trials. Normalized sEMG signals are therefore presented as a percentage value (%) of muscle activity relative to maximum value observed during canter. Reference voluntary contractions represented submaximal contractions that were generally lower than those observed during jump trials, so normalized sEMG signals from jump are generally greater than 100% of the reference value.

Normalization using an RVC is reliant on a maximal amplitude derived from the sEMG signal, which is vulnerable to many sources of variability [49]. Although appropriate signal detection and post-processing techniques can mitigate this variability [49], it is important to detect and remove any outliers from the dataset to ensure that sEMG signals normalized to a value that accurately reflects the maximal muscular effort observed for the studied task/gait. This will improve the reliability of data used for gait analysis without affecting natural biological variations [64]. Thus, prior to normalization of sEMG data, outliers in peak amplitude data from canter were detected and removed, by setting upper and lower outlier limits as 2 standard deviations outside of the mean peak amplitude values within each subject, muscle and task [65]. Peak amplitude data from A1 and jump strides were also scanned for outlier strides prior to normalization and further analysis.

### 2.6. Statistical Analysis

To analyze potential differences in movement and muscle function, horses were split into subgroups based on their ability to elevate the CM during jump suspension (Z_CM_). Z_CM_ was chosen as a discriminative, objective performance indicator because it is documented and generally accepted as the main determinant of jumping capacity/success [7,27]. For comparative analyses, the mean Z_CM_ was split according to the 33.3 and 66.6 percentiles using Z_CM_ data from each horse, which was used to split horses into three subgroups: horses with the highest Z_CM_ (High_CM_) (*n* = 5, standing height = 162.8 ± 7.3 cm, Z_CM_ > 0.50 m), horses with the lowest Z_CM_ (Low_CM_) (*n* = 5, standing height = 158.4 ± 6.6 cm, Z_CM_ < 0.37 m) and the remaining horses forming the intermediate group (Int._CM_) (*n* = 7, standing height = 155.3 ± 8.9 cm).

Descriptive statistics (mean ± sd) were calculated for discrete kinematic and sEMG activity timing variables within each horse, stride (A1, jump stride) and limb (LdH/LdF, TrH/TrF) combination. Between-group differences within each stride and limb combination, were analyzed using one-way between-subjects ANOVAs. Post-hoc comparisons were investigated with a Bonferroni correction when significant main effects were identified. Differences between limbs (TrH, LdH) were tested using paired samples *t*-tests. Correlations were calculated to examine potential relationships between muscle function and performance indicators. Pearson correlation coefficients were calculated between the discrete kinematic and sEMG variables that showed significant between-group differences. Analyses were conducted using IBM SPSS Statistics for Windows version 27.0 (IBM Corp., Armonk, NY, USA). Values of *p* < 0.05 were considered significant and ANOVA effect sizes were established using partial eta^2^.

Between-group differences in time and amplitude-normalized sEMG waveforms from each muscle, stride (A1 and jump stride, 101 data points per cycle) and limb (LdH, TrH) combination were examined using one-dimensional SPM, conducted in a hierarchical manner akin to a one-way between-subjects ANOVA, followed in the event of a significant main effect, by post-hoc independent-samples *t*-tests. SPM analysis was conducted in MATLAB 2019a (MathWorks, Natick, MA, USA) and values of *p* < 0.05 were considered significant.

## 3. Results

### 3.1. Kinematic Examination of Equestrian Performance Indicators

Descriptive statistics (mean ± sd) for equestrian-derived performance indicators and associated kinematic outcome measures are presented in Table 1. Data are organized under the three broad themes of joint articulation, impulsion and engagement. For each kinematic variable, significant (*p* < 0.05) between-limb and between-group differences are presented (Table 1). Results from pairwise comparisons, where a significant (*p* < 0.05) main effect was found between groups, are presented in Table 1 as corresponding superscripts and in more detail in Supplementary Table S2 as mean difference, *p*-values and 95% confidence intervals. The time–angle/segment and time–course curves, used to calculate discrete values for kinematic outcome measures, are illustrated in Supplementary Figures S1 and S2.

Kinematic outcome measures used to evaluate equestrian preferences for joint articulation revealed that High_CM_ horses reached maximum TrF scapula inclination and LdH shortening significantly earlier in the jump stride than Low_CM_ horses (*p* < 0.05). Int._CM_ horses exhibited significantly greater TrH and LdH retraction and LdH radius angle than Low_CM_ horses (*p* < 0.05). Within the engaged theme, kinematic variables showed that High_CM_ horses reached maximum TrH shortening, TrH hock and TrH stifle flexion significantly earlier than Low_CM_ (*p* < 0.05). The LdH stifle joint also reached maximum flexion significantly earlier in High_CM_ horses compared to Low_CM_ horses.

**Figure 4:**
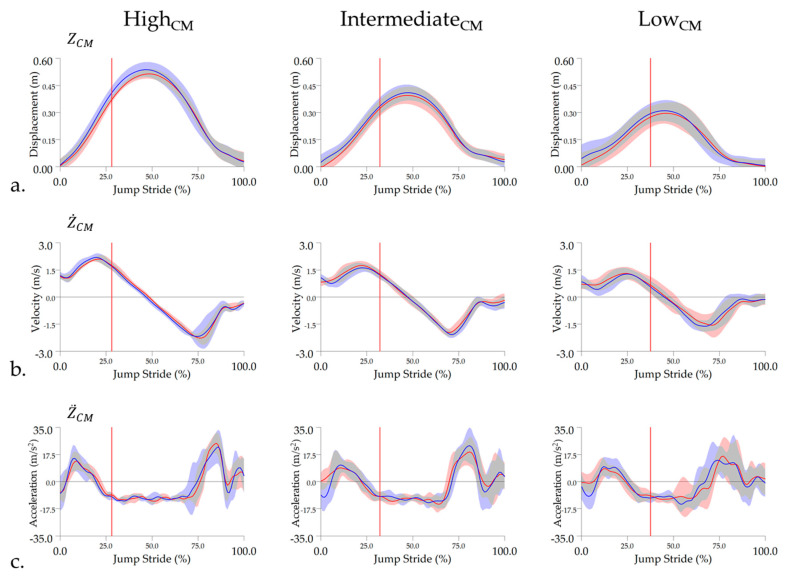
Mean and standard deviation time–course data for vertical (**a**) displacement (Z_CM_), (**b**) velocity (${\dot{Z}}_{CM}$), and (**c**) acceleration (${\ddot{Z}}_{CM}$) of the center of mass marker. Data are presented in separate columns for High_CM_, Intermediate_CM_, and Low_CM_ groups and are normalized to jump stride duration. Red vertical lines represent the average hindlimb lift-off event within each group. Mean data are presented for leading hindlimb (LdH) (red line) and trailing hindlimb (TrH) (blue line), with shaded areas representing the standard deviation for each limb.

Significant main effects were found for the majority of kinematic outcome measures under the impulsion theme (Table 1). High_CM_ horses approached the fence significantly faster than Low_CM_ horses when the measured (right) hindlimb functioned as TrH and exhibited significantly shorter LdH A1 stance duration and significantly lower LdH and TrH jump stride duty factor than Low_CM_ horses (*p* < 0.05). Low_CM_ horses exhibited significantly longer forelimb stance durations than High_CM_ and Int._CM_ groups (*p* < 0.05), but this was only significant between Int._CM_ and Low_CM_ for TrF (*p* = 0.04) (Table S2). High_CM_ horses achieved significantly greater CM elevation (Z_CM_) than Int._CM_ (*p* < 0.05) and Low_CM_ groups (*p* < 0.001), with Int._CM_ horses also showing significantly greater Z_CM_ than Low_CM_ horses (*p* < 0.05). High_CM_ and Int._CM_ horses also produced significantly greater ${\dot{Z}}_{CM}$ than Low_CM_ horses (*p* < 0.05), but this was only significant between Int._CM_ and Low_CM_ when the measured (right) hindlimb functioned as LdH (*p* = 0.03) (Table S2). When the measured (right) hindlimb functioned as TrH, High_CM_ horses reached ${\dot{Z}}_{CM}$ and ${\ddot{Z}}_{CM}$ significantly earlier in the jump stride than Low_CM_ horses (*p* < 0.05). Time–course curves for Z_CM_, ${\dot{Z}}_{CM}$ and ${\ddot{Z}}_{CM}$ are illustrated in Figure 4.

Across all kinematic variables, between-limb differences were only found for A1 stride duty factor (*p* = 0.02), jump stride velocity (*p* = 0.03) and stifle joint flexion (*p* = 0.02) (Table 1). A1 stride duty factor and jump stride velocity were significantly greater when the measured (right) hindlimb functioned as LdH compared to TrH (*p* < 0.05), while stifle joint flexion was significantly greater in the TrH.

### 3.2. Muscle Activity Patterns during Jumping

**Figure 5:**
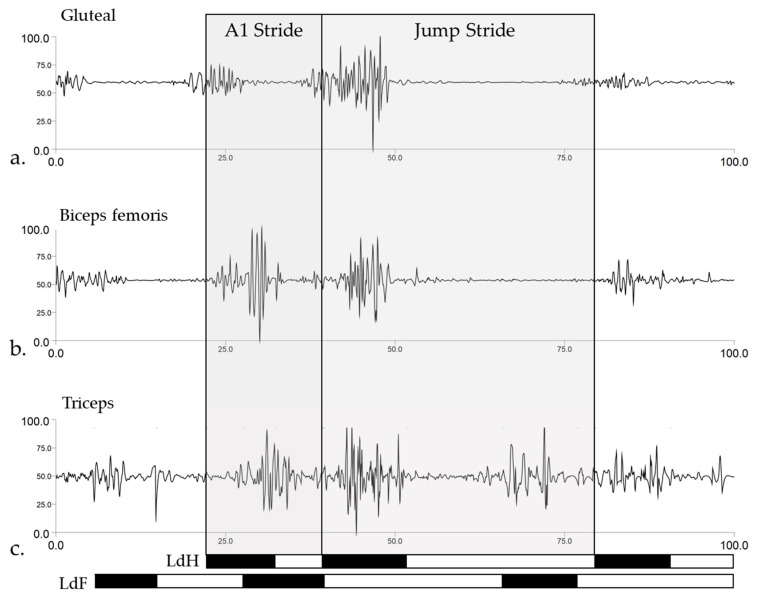
Phasic activity patterns of (**a**) gluteal, (**b**) biceps femoris, and (**c**) triceps muscles from a representative Intermediate_CM_ horse and jump trial. Approach (A1) stride and jump stride are labelled and illustrated as grey shaded boxes. sEMG signals are direct current (DC)-offset removed and high-pass filtered (Butterworth 4th order, 40 Hz cut-off frequency). Bottom bars represent stance (black shaded areas) and swing (white shaded areas) phases for the right hindlimb and forelimb that acted as leading hindlimb (LdH) and leading forelimb (LdF), respectively, during this jump trial.

An overview of the general phasic activity patterns of gluteal, biceps femoris and triceps muscles and right forelimb and hindlimb temporal data from one representative Int._CM_ horse and jumping trial is presented in Figure 5. High-pass filtered sEMG signals are presented to give the reader an indication of sEMG signal quality and activity pattern during jumping trials, as the following subsections will employ enveloped, group-averaged sEMG signals to present between-group differences for separate A1 and jump strides. Figure 5 illustrates the muscle activation bursts that are described in the preceding sections, as well as the marked reduction in activity across all muscles during jump suspension.

Although there were no significant between-group differences when analyzing the sEMG waveform and timing data solely by group using SPM (*p* > 0.05) (Supplementary Figures S3–S5), important pairwise differences were found for discrete sEMG timings variables when muscle, stride, and limb factors were included in the ANOVA analysis. Descriptive statistics (mean ± sd) for sEMG timing variables are presented in Table 2. Between-limb and between-group differences for each timing variable are presented as *p*-value and effect sizes (eta^2^) (Table 2). Results from pairwise comparisons, where a significant (*p* < 0.05) main effect was found between groups, are presented in in Table 1 as corresponding superscripts and in Supplementary Table S3 as mean difference, *p*-values and 95% confidence intervals.

#### 3.2.1. Middle Gluteal

**Figure 6:**
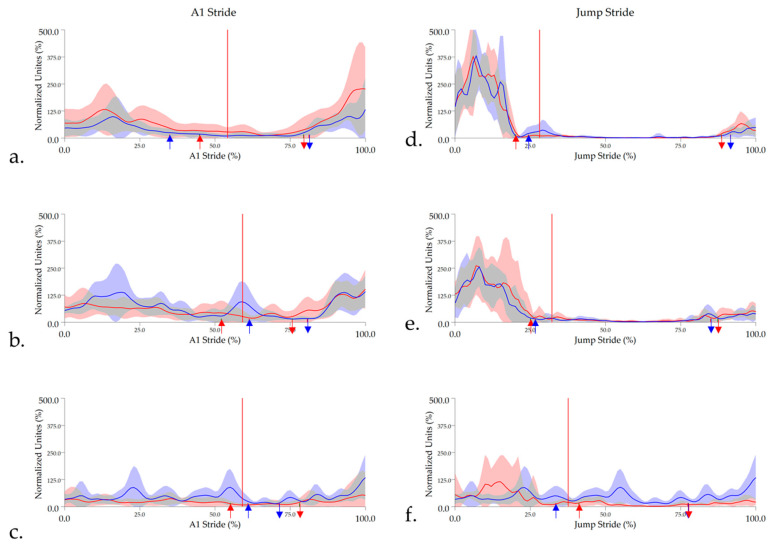
Mean and standard deviation time and amplitude-normalized, linear enveloped sEMG signals from gluteal for (**a**) High_CM_, (**b**) Int_CM_, and (**c**) Low_CM_ horses during approach (A1) stride and (**d**) High_CM_, (**e**) Int_CM_, and (**f**) Low_CM_ during jump stride. sEMG signals are DC-offset, high-pass filtered (40 Hz cut-off) and low-pass filtered (25 Hz). Mean data are presented for leading hindlimb (LdH) (red line) and trailing hindlimb (TrH) (blue line), with shaded areas representing the standard deviation for each limb. Arrows represent the mean gluteal activity onset (downward arrows) and offset (upward arrows) events for LdH (red arrows) and TrH (blue arrows). Red vertical lines represent the average hindlimb lift-off event within each group.

Gluteal activity onset occurred prior to the hindlimb impact that initiated A1 stride and remained active for the majority of hindlimb stance phase. After a short, quiet period, the muscle became active at approximately 75–80% of the A1 stride cycle, just prior to hindlimb impact at take-off. A significant main effect was found for the TrH activity offset event, with High_CM_ horses showing significantly earlier offset of gluteal activity than Low_CM_ and Int._CM_ horses (*p* < 0.05), as reflected in the shorter, but non-significant activity duration. During jump stride, the gluteal remained active for the majority or entirety of hindlimb stance phase at take-off until approximately 25–35% of the stride cycle. The gluteal remained largely quiet during jump suspension, becoming active just prior to hindlimb impact at landing (approximately 75–90% of stride cycle). High_CM_ horses had significantly shorter gluteal activity duration across both limbs than Low_CM_ horses *p* < 0.05) and a significantly later TrH activity onset event prior to landing (*p* = 0.02), which approached significance for LdH (*p* = 0.05) (Table 2 and Table S3). Group-averaged, enveloped sEMG signals and activity onset/offset events from the gluteal muscle are presented in Figure 6.

#### 3.2.2. Biceps Femoris

**Figure 7:**
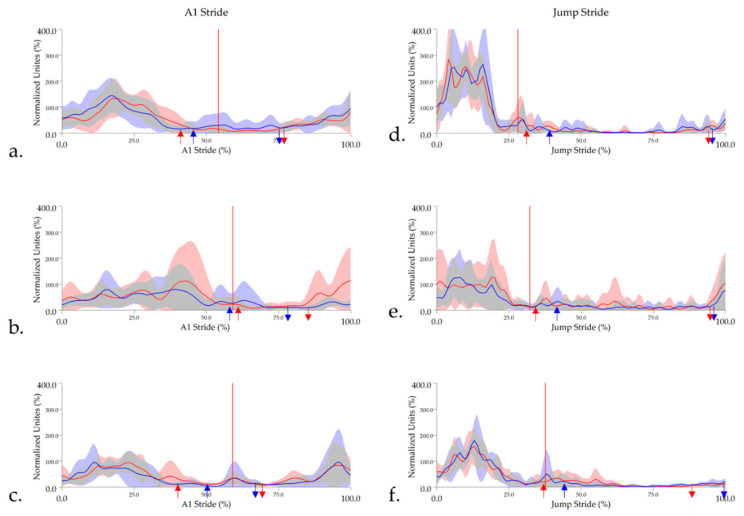
Mean and standard deviation time and amplitude-normalized, linear enveloped sEMG signals from biceps femoris for (**a**) High_CM_, (**b**) Int_CM_, and (**c**) Low_CM_ horses during approach (A1) stride and (**d**) High_CM_, (**e**) Int_CM_, and (**f**) Low_CM_ during jump stride. sEMG signals are DC-offset, high-pass filtered (40 Hz cut-off) and low-pass filtered (25 Hz). Mean data are presented for leading hindlimb (LdH) (red line) and trailing hindlimb (TrH) (blue line), with shaded areas representing the standard deviation for each limb. Arrows represent the mean biceps femoris activity onset (downward arrows) and offset (upward arrows) events for LdH (red arrows) and TrH (blue arrows). Red vertical lines represent the average hindlimb lift-off event within each group.

Biceps femoris displayed similar activity pattern and subsequent activity timings to the gluteal muscle. A significant main effect was found for activity offset during A1 stride, which occurred at approximately 40% in High_CM_ and Low_CM_ horses but was active for significantly longer in the TrH of Int._CM_ horses compared to High_CM_ horses (*p* = 0.04) (Table 2 and Table S3). The same trend was observed for LdH, but this was non-significant (*p* = 0.18) (Table 2). Significant main effects were not observed for biceps femoris activity timings during jump stride. Biceps femoris activity offset occurred significantly later (*p* < 0.05) in the LdH during both A1 and jump stride cycles (Table 2). Group-averaged, enveloped sEMG signals and activity onset/offset events for biceps femoris are presented in Figure 7.

#### 3.2.3. Triceps Brachii

**Figure 8:**
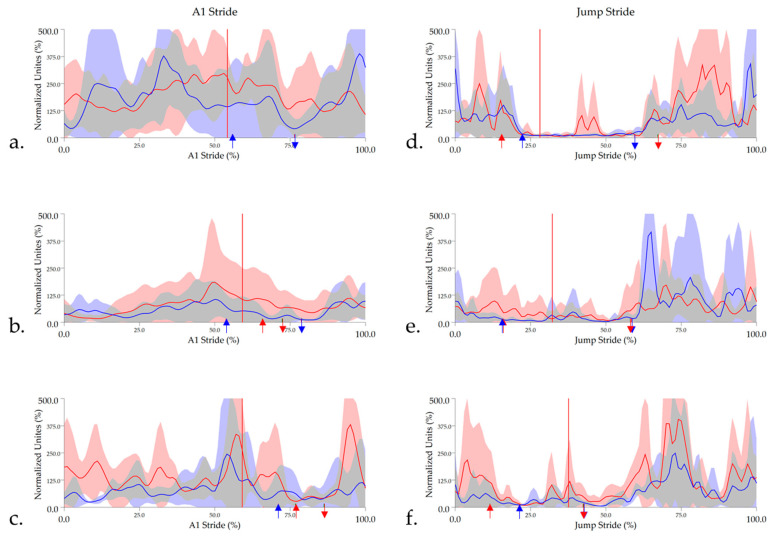
Mean and standard deviation time and amplitude-normalized, linear enveloped sEMG signals for triceps in (**a**) High_CM_, (**b**) Int_CM_, and (**c**) Low_CM_ horses during A1 stride and (**d**) High_CM_, (**e**) Int_CM_, and (**f**) Low_CM_ during jump stride. sEMG signals are DC-offset, high-pass filtered (40 Hz cut-off) and low-pass filtered (25 Hz). Mean data are presented for leading forelimb (LdF) (red line) and trailing forelimb (TrF) (blue line), with shaded areas representing the standard deviation for each limb. Arrows represent the mean triceps activity onset (downward arrows) and offset (upward arrows) events for LdF (red arrows) and TrF (blue arrows). Red vertical lines represent the average hindlimb lift-off event within each group.

Triceps muscle activity patterns exhibited the greatest variation across the muscles studied, particularly during A1 stride, which likely accounted for non-significant between-group differences for triceps activity during A1 and jump stride. During A1 stride, a double burst pattern was observed, with the first occurring for the majority or entirely of forelimb stance (until approx. 65–75% stride duration) and the second occurring between forelimb lift-off and hindlimb impact at take-off (approximately 75–80% stride duration). In most trials, the amplitude between bursts did not decrease enough to be identified as activity offset, so triceps activity duration varied from approximately 65–100% of stride duration. In 35.3% of trials, the triceps was active for 100% of A1 stride, with High_CM_ horses accounting for 23.5% of these trials, hence the missing data for onset/offset events in A1 stride in Table 2. During jump stride, the triceps was generally active for longer than gluteal and biceps femoris muscles, largely due to earlier activity onset at approximately 60% of jump stride, which coincided with forelimb impact at landing. However, in contrast to hindlimb muscles, the triceps activation was shorter during take-off, with activity offset occurring between 10 and 20% of jump stride. Group-averaged, enveloped sEMG signals and activity onset/offset events for triceps are presented in Figure 8.

### 3.3. Relationships between Significant Muscle Function and Jumping Performance Indicators

To ensure that the between-group differences were linked to overall jump technique/performance, correlations between each significant kinematic variable and the discriminative performance variable, Z_CM_, were calculated and are presented in Supplementary Table S4. All kinematic variables with between-group differences were found to be significantly correlated with Z_CM_ (r > 0.55, *p* < 0.05), except LdF maximum radius angle (*p* > 0.05) and TrH maximum hindlimb retraction angle which approached a significant correlation with TrH Z_CM_ (r = 0.46, *p* = 0.07) (Table S4) and was thus carried forward for evaluation against muscle function. Correlations between significant hindlimb sEMG timings variables and kinematic variables are presented in Table 3. Correlations between triceps activity and kinematic performance measures associated with the forelimb were not calculated due to non-significant between-group differences in this muscle.

## 4. Discussion

This study is the first to combine kinematic with sEMG data during equine jumping where movement and muscle activation strategies are compared for horses grouped according to a fundamental determinant of jumping capacity and success. The following sections focus on the significant differences observed in movement and muscle activation strategies between groups, how these differences may be facilitated through differing neuromuscular strategies and whether relationships between movement and muscle activity can be used to determine indicators of performance for the selection and training of jumping horses.

### 4.1. Movement and Hindlimb Muscle Activation Strategies That Facilitate Impulsion and Engagement Represent Key Jumping Performance Indicators

The Fédération Equestre Internationale defines impulsion as upward thrust or the release of energy stored by engagement; achieved through controlled muscular power in the hindquarters, enabling increased hindlimb: protraction, joint flexion and subsequent shortening [54]. From a biomechanical perspective, this agrees with the known relationship between the amount of positive work generated by the hindlimb and its total length change, through compression/flexion of the hindlimb joints during stance [39]. Further, the mechanical energy required to execute the jump is produced during take-off [15,17,25,39,66], with the vertical impulse of hindlimb ground reaction forces governing the initial velocity of the CM, which in turn determines its ballistic flight during jump suspension [25,27,67]. A horse’s jumping capacity is therefore determined by vertical impulse at take-off, which is influenced by approach speed, stride duration and muscular force production [67]. Findings from this study agree with this, as A1 stride velocity, jump stride duty factor, Z_CM,_ ${\dot{Z}}_{CM}$*,* and hindlimb muscle activation timings differed significantly between High_CM_ and Low_CM_ horses. Between-group differences were not observed for overall shortening of the hindlimb segment or joint flexion angles (hip, stifle, hock), but rather the time at which maximum joint flexion and shortening events occurred. High_CM_ horses displayed more rapid flexion of the TrH hock and stifle joints and subsequent shortening of the hindlimb segment at take-off than Low_CM_ horses. This finding suggests that High_CM_ horses experience more rapid stretching of the elastic soft tissues, which may also result in greater elastic rebound and a more efficient jumping technique. Thus, greater approach stride velocity, shorter hindlimb ground contact and faster hindlimb compression at take-off and greater elevation and vertical velocity of the CM during the jump stride show the enhanced neuromuscular control (motor skill) of High_CM_ horses for jumping. These findings also provide objective support for the importance of equestrian performance indicators related to impulsion and engagement for evaluating overall jumping performance.

Studies have reported that maximum vertical displacement of the CM during jump suspension is positively correlated with vertical velocity of the CM during take-off [6,7,14,19,26]. Findings from this study agree with this, as Low_CM_ horses displayed significantly lower ${\dot{Z}}_{CM}$ than horses with the highest Z_CM_ and a strong positive correlation was observed between both variables (Table S4). Increased generation of ${\dot{Z}}_{CM}$ at take-off has also been linked to shorter HL stance duration and higher peak vertical acceleration of CM (${\ddot{Z}}_{CM}$), which characterizes the ability to generate greater vertical force over a shorter contact period [26]. This is also largely in accordance with findings from this study, as High_CM_ horses exhibited significantly shorter jump stride duty factor and significantly earlier peaks in ${\dot{Z}}_{CM}$ and ${\ddot{Z}}_{CM}$ in the trailing hindlimb than Low_CM_ horses. In accordance with Barrey and Galloux [25], High_CM_ horses were also found to approach the fence significantly faster than Low_CM_ horses, which indicates a more efficient conversion of horizontal velocity into vertical velocity at take-off. High_CM_ horses also exhibited shorter hindlimb stance duration, which is known to decrease with increasing speed [68,69].

#### Relationships between Hindlimb Muscle Activation and Kinematic Measures of Impulsion and Engagement

The middle gluteal and biceps femoris muscles function to extend and abduct the hip, with the biceps also functioning to flex the stifle and extend the hock joint during swing phase [70]. Previous studies have made inferences regarding equine muscle function during jump take-off [15,39,66], but these have not previously been substantiated by sEMG data. At take-off, positive work performed by the hindlimb is related to the increase in total limb length from hoof impact and lift-off [39]. At the beginning of hindlimb stance, the limbs are shortened as hindlimb joints experience flexion under the control of extensor musculature, which contract eccentrically to counteract external forces [15,39,53,66,71]. Thus, the hindlimb has been reported to absorb energy (or create net negative power) by active and passive elements of the muscle–tendon units during the initial 40% of stance [71]. In the second half of stance phase, power is generated as all hindlimb joints extend, which is aided by concentric contraction of extensor musculature and the release of elastic energy that was stored during limb loading [15,39,53,71]. It is important to note that there are inherent issues with defining muscle contraction type from sEMG signals alone, especially for dynamic movements where variation in joint angular velocities, muscle forces and the increased risk of electrode movement distort the relationship between muscle force and sEMG amplitude [72]. However, sEMG signal activation patterns and timings from gluteal and biceps femoris and hindlimb kinematic data largely support these descriptions for hindlimb extensor muscle function during jump take-off.

sEMG onset/offset events revealed differences in the activation strategies of the studied hindlimb muscles in horses with varying jump technique, particularly in the gluteal muscle during jump stride. Gluteal activation in High_CM_ horses was characterized by significant reductions in activity duration compared to Low_CM_ horses, which was achieved through earlier activity offset at jump take-off and significantly later activity onset at landing. It has been suggested that greater hindlimb muscular strength and increased motor skill allows more experienced/better-performing jumping horses to generate the vertical impulse and explosive power required to elevate the CM over a faster approach velocity and reduced contact time at take-off [67]. Gluteal activity duration had a strong negative correlation with Z_CM_ and ${\dot{Z}}_{CM},$ and a strong positive correlation with A1 stride velocity, jump stride duty factor and timing of maximum hindlimb shortening, which supports the suggested relationship between muscular strength and jumping performance [67]. Taken together, between-group differences in gluteal activity timings and the significant correlations with these kinematic variables suggest that, in comparison with Low_CM_ horses, High_CM_ horses are able to approach the fence faster and produce the muscular force required to elevate the CM more rapidly, which is achieved by rapid gluteal contraction and compression of the hindlimb over a shorter contact time at take-off.

Biceps femoris activity was similar across groups during jumping, with activity offset in A1 stride representing the only significant difference between groups. The largely non-significant differences in biceps femoris activation across groups and the lack of correlations with equestrian performance indicators suggest that gluteal muscle plays a greater role for facilitating better jumping technique/performance. Thus, the ability of the horse to produce rapid bursts of power in the middle gluteal muscles during approach and take-off, which increase vertical displacement and velocity of the CM, are important indicators of superior jumping performance and represent objective indicators of a horse’s capacity for executing larger fences or its “scope”. These findings also suggest that power development, particularly for the middle gluteal muscles, is a worthwhile training objective for jumping horses.

### 4.2. Forelimb Joint Articulation and Triceps Muscle Activation Strategies Do Not Differentiate Jumping Performance over Submaximal Fences

Findings from St. George et al. [11] revealed that equestrians deemed forelimb and hindlimb joint articulation as important for distinguishing good-quality jump technique. Previous studies agree with this, reporting that an efficient jump technique in better-performing horses is characterized by a lower Z_CM_, greater shortening of the forelimb and hindlimb segments and greater hindlimb retraction to aid fence clearance [6,7,26,73]. Direct comparisons with these studies are difficult, as horses in this study were generally older with more jumping experience and were not grouped based on competitive jumping performance/capacity. However, forelimb and hindlimb joint articulation variables in this study were similar, and generally non-significant, between groups. Thus, although aesthetically pleasing and functionally important for reducing the risk of jumping faults, findings suggest that equestrian-derived performance indicators related to forelimb and hindlimb joint articulation during the flight phase do not differentiate between ridden jumping technique when evaluated over submaximal fences. Fence height has been shown to affect jumping kinematics [19,22], so future studies are required to examine performance indicators and muscle function over larger fences, which may highlight additional performance indicators for forelimb and hindlimb movement. Further research in this area will also provide a better understanding of intersegmental coordination patterns and how they affect not just jumping height but also how they interact with CM height to ensure fence clearance.

#### Relationships between Triceps Muscle Activation and Kinematic Measures Forelimb Joint/Segment Movement

Interestingly, no significant differences were found for forelimb kinematic variables, except for the timing of peak TrF scapula inclination, maximum LdF radius angle and forelimb stance duration in A1 stride. The triceps was employed to examine forelimb muscle function during jumping and functions to extend the elbow and flex the shoulder, working as an antagonist to the biceps brachii to stabilize the shoulder and elbow joints [74]. It has been proposed that during forelimb stance in A1 stride, the forelimbs function as passive springs, based on the idea that kinetic energy is initially stored in tendons and released during push-off to generate kinetic and potential energy [75]. However, Bobbert and Santamaria [39] showed that this analogy is only partially true and that activation of proximal forelimb muscles, specifically the triceps, must contribute to the regeneration of energy lost through dissipation during forelimb push-off, but that electromyography data were required to provide further evidence of this. Indeed, triceps activation throughout most of A1 stride and the large normalized values observed, provide support for the energetic demands of forelimb musculature during the A1 stride, as proposed by Bobbert and Santamaria [39]. As significant differences in forelimb kinematics and triceps muscle activity timings and amplitude were not observed between groups, it appears that the jump task was performed in a similar manner in the forelimbs, independent of skill. This may, in part, relate to using set distances between fences that provide more consistent take-off distances for less skilled horses and riders and supports the use of such methods in training.

### 4.3. Study Limitations and Additional Considerations

Differences in normalized sEMG amplitude across A1 and jump stride cycles were explored using SPM, but significant differences between groups were not observed, which is likely related to the between- and within-group variation observed in normalized sEMG signal amplitudes (Figure 6, Figure 7 and Figure 8). Alpha is more tightly controlled using SPM than in traditional statistical analysis and variation in equine kinematic and kinetic data have been shown to affect the level of significance using SPM [76]. sEMG signal amplitude is sensitive to several internal and external factors [49], so appropriate normalization techniques must be applied to reduce between and within-subject variation and to allow comparisons between subjects, muscles and trials [77,78]. In equine subjects, obtaining a maximum voluntary contraction (MVC) is not possible, but normalization to a submaximal RVC has been shown to improve sensitivity and accuracy of equine gait analysis [32]. In this study normalization to the maximum signal observed during canter permitted examination of the meaningful proportional change in muscle activity between the related activities of canter and jump. However, EMG amplitude is sensitive to changes in the velocity of movement [79], which could not be standardized in this study for canter and especially for jumping activities, where stride velocities differ significantly between approach and jump strides [62]. Further, data were only collected from the right forelimb and hindlimb, so we also cannot rule out the possibility of laterality effects on canter variables from left and right leads [80]. Thus, differences in velocity and a preference/muscular asymmetry between left and right canter lead may have influenced the variation observed in normalized sEMG signals from jumping trials. Future studies may wish to explore alternative normalization techniques for the equine jump technique, but we do not recommend normalization to an RVC obtained during jump trials, due to variation in exertion and subsequent issues with reproducibility of this activity [17]. Variation in approach speed was also not corrected for statistically because of the between-subjects study design and the method for grouping horses based on Z_CM_, which is partially influenced by approach speed [67]. As such, the statistical analysis techniques employed in this study may have limitations when compared to others, given the known effects of variation in locomotor speed on muscle activity patterns and kinematics [79]. However, approach speed was considered at least partially responsible for the measured difference in jump techniques, which we aimed to quantify, and the subsequent grouping of horses. Further, we endeavored to minimize rider influence and variation in approach speed by employing a grid line to standardize take-off. Thus, statistical correction for speed should be a consideration for future studies to determine variables that best predict jumping capacity but was not considered appropriate for the current study design.

Z_CM_ was chosen as the single, discriminative variable to categorize horses based on variations in jump technique. In this study, a reference point on the trunk, which coincided with the CM location, was calculated using a modified version of the method described by Bogert et al. [15] for jumping horses. Z_CM_ values from the standing horse and normalized values during jump suspension were similar to those reported in previous studies employing a comparable fence of 1.05 m [14,26]. Thus, the method for calculating the CM reference point was considered sufficiently accurate. From a biomechanical perspective, the horse’s ability to raise and rotate the body around the CM represents the main determinant of jumping capacity/success and has been identified as a discriminative predictor of jumping performance [6,7,16,25,26,27]. However, jumping performance is multifactorial in nature and cannot be quantified by movement alone. Thus, further work is required to assess the effectiveness of objective performance indicators from this study in relation to longitudinal competitive performance in a large sample of horse/rider combinations.

It is important to note that significant differences were not observed when comparing the function of the right hindlimb as leading or trailing limb for many of the study variables. These findings are not surprising for the jump stride, as the hindlimbs are known to function relatively symmetrically at take-off, especially as fence height increases [53]. Collecting data from the right limbs on both canter leads enabled a direct comparison of movement and muscle activity when they functioned as either LdH and LdF or TrH and TrF. However, the measurement of one (right) limb required that stride splitting was conducted using either LdH or TrH impact events, dependent on the measured canter lead. Thus, comparisons in temporal stride characteristics between leading and trailing limbs were confounded by the stride splitting method.

Finally, as an additional consideration, this study was heavily focused on differences in temporal measures of muscular activity. Most equine sEMG studies have generally employed kinematically derived gait events to provide descriptive data on muscle activity patterns [33,42], but muscle activation timings are a requirement for developing a comprehensive understanding of equine muscle function. Unfortunately, there is no consensus amongst sEMG researchers involved in human studies regarding an optimal sEMG onset/offset detection method [81], so the method described in this study was derived from various human sEMG event detection methods that lent themselves to the unique challenges associated with detecting and processing equine sEMG data. This study is the first to describe an adapted double-threshold method [58,59] for equine sEMG data, offering a simple, flexible approach for accurate onset/offset detection, where the timing and amplitude thresholds can be adapted (within reason) to the specific muscle and activity under investigation. A degree of adaptability in these methods is recommended in human research, where fixed, a priori timing and amplitude thresholds have been shown to result in erroneous onset/offset detection [82]. Many human sEMG onset/offset detection methods rely on an amplitude threshold derived from resting muscle activity [81]. The benefit of this method is that the threshold is based on a statistical deviation from the baseline/resting value of a specific muscle [83], but there is an implicit assumption that EMG amplitude is sufficiently greater during isokinetic contractions than at rest. The assumption that muscles are at “rest” in a standing horse of approximately 500 kg may not be appropriate, especially in this study where standing trials were conducted with a mounted rider. Further, it is not possible to instruct equine subjects to rest/relax when collecting baseline muscle activity data, as is often done in human subjects [81]. Thus, in accordance with [60], we recommend defining the amplitude threshold as a percentage of the peak amplitude value of each individual signal. A 5% or 10% amplitude threshold detected onset/offset events consistently and accurately for triceps, biceps femoris and gluteal, but could be adapted for postural muscles which generally display higher baseline activity [34]. It is, however, important that the amplitude threshold is not increased to compensate for poor signal quality. In accordance with [61], defining the timing threshold as 5% of the average gait cycle duration is recommended to allow correlation across equine gaits/activities that exhibit differing stride durations and velocities. This original, flexible method for equine sEMG activity detection performs well, as illustrated by its ability to detect significant differences in muscle activity patterns between groups.

## 5. Conclusions

In this study, sEMG data offered original insight into fundamental muscle activity patterns of selected superficial equine muscles during a submaximal jumping effort, which agreed with literature on the functional role of equine muscles during jumping. Differences in muscle activation were most pronounced in the gluteal muscle during jump stride, where shorter contractions at take-off were significantly correlated with higher and more rapid vertical displacement and vertical velocity of the CM trajectory, a faster approach, shorter hindlimb stance duration at take-off and more rapid shortening of the hindlimb at take-off. Thus, horses with a greater capacity to elevate the CM during jump suspension displayed a greater ability to generate muscular power and vertical impulse rapidly during jump take-off, supporting the hypotheses that horses with the greatest CM elevation exhibit desirable kinematic traits that are associated with muscle activation patterns, and differ significantly from horses with lower CM elevation. These findings provide objective support for equestrian preferences related to the generation of engagement, impulsion and hindlimb muscle power when selecting jumping horses and justify their prioritization as objective performance indicators for the sport of equine jumping. Results also suggest that less emphasis is placed on equestrian-derived performance indicators related to the forelimb during the approach stride and forelimb and hindlimb joint articulation during flight, which did not differentiate jumping performance over submaximal fences. These indicators should therefore be considered as secondary to equestrian preferences for impulsion and engagement. This study has also highlighted the importance of power-training exercises within jump training programs, which could support the development of improved jump technique and performance.

## Figures and Tables

**Table 1:** Descriptive statistics (mean ± standard deviation) for kinematic variables within each subgroup of horses. Between-group differences are presented for each variable as *p*-values and effect sizes (eta^2^). Within each row, significant main effects (*p* < 0.05) for between-group differences are denoted by bold text and significant differences (*p* < 0.05) between groups are represented by corresponding superscripts (^a–c^). Significant differences (*p* < 0.05) between limbs are denoted by grey shaded cells.

Theme	Kinematic Variable	Limb	Group			*p*	eta^2^
			High_CM_	Int._CM_	Low_CM_		
Joint Articulation	Max shoulder flex (°)	TrF	10.8 ± 2.2	11.4 ± 6.0	8.6 ± 3.3	0.58	0.08
		LdF	12.3 ± 1.8	11.7 ± 3.9	8.8 ± 3.1	0.22	0.20
	Max shoulder flex time (% jump stride)	TrF	19.2 ± 2.1	20.8 ± 9.9	23.6 ± 6.0	0.67	0.06
		LdF	17.0 ± 4.5	21.5 ± 4.2	25.2 ± 6.2	0.18	0.27
	Max scapula angle (°)	TrF	55.3 ± 6.7	49.3 ± 8.3	44.2 ± 8.3	0.15	0.25
		LdF	53.2 ± 3.2	49.1 ± 8.5	40.8 ± 8.9	0.06	0.34
	Max scapula angle time (% jump stride)	TrF	**19.1 ± 0.7 ^a^**	**23.1 ± 3.5**	**23.6 ± 1.1 ^a^**	**0.03**	**0.45**
		LdF	19.3 ± 1.2	24.8 ± 4.7	23.3 ± 3.4	0.10	0.29
	Max FL shortening (m)	TrF	−0.5 ± 0.0	−0.5 ± 0.0	−0.5 ± 0.0	0.26	0.18
		LdF	−0.5 ± 0.1	−0.5 ± 0.0	−0.5 ± 0.0	0.41	0.13
	Max FL shortening time (% jump stride)	TrF	31.5 ± 4.7	27.6 ± 5.0	25.9 ± 8.3	0.43	0.13
		LdF	32.5 ± 2.1	28.0 ± 4.0	29.1 ± 11.7	0.77	0.05
	Max carpus flex (°)	TrF	118.5 ± 9.0	128.3 ± 7.2	124.4 ± 14.9	0.37	0.17
		LdF	117.4 ± 5.6	130.7 ± 9.8	126.3 ± 16.1	0.21	0.21
	Max carpus flex time (% jump stride)	TrF	28.6 ± 3.9	24.8 ± 1.5	25.6 ± 8.3	0.55	0.11
		LdF	31.1 ± 2.2	28.2 ± 4.5	23.3 ± 11.8	0.43	0.14
	Max elbow flex (°)	TrF	81.0 ± 3.8	85.0 ± 7.9	87.0 ± 8.6	0.51	0.12
		LdF	78.0 ± 5.8	85.9 ± 6.8	81.0 ± 10.6	0.25	0.18
	Max elbow flex time (% jump stride)	TrF	31.9 ± 13.0	35.5 ± 5.9	26.2 ± 9.5	0.38	0.18
		LdF	42.1 ± 2.1	37.6 ± 6.1	38.8 ± 6.6	0.67	0.08
	Max radius angle (°)	TrF	76.6 ± 5.0	82.8 ± 8.3	75.8 ± 7.7	0.26	0.20
		LdF	**75.3 ± 7.5**	**82.2 ± 4.8 ^a^**	**71.9 ± 7.5 ^a^**	**0.04**	**0.37**
	Max radius angle time (% jump stride)	TrF	41.0 ± 3.7	36.0 ± 8.4	27.3 ± 12.3	0.13	0.31
		LdF	39.9 ± 10.4	40.0 ± 6.9	41.9 ± 7.2	0.91	0.02
	Max HL shortening (m)	TrH	−0.4 ± 0.1	−0.4 ± 0.1	−0.4 ± 0.1	0.35	0.15
		LdH	−0.4 ± 0.0	−0.4 ± 0.1	−0.4 ± 0.1	0.41	0.12
	Max HL shortening time (% jump stride)	TrH	79.6 ± 7.1	80.6 ± 7.0	84.3 ± 4.4	0.50	0.11
		LdH	**68.5 ± 11.8 ^a^**	**80.4 ± 6.0**	**83.7 ± 4.5 ^a^**	**0.02**	**0.44**
	Max HL retraction (°)	TrH	**−50.2 ± 0.9**	**−46.0 ± 2.7 ^a^**	**−52.5 ± 3.4 ^a^**	**0.00**	**0.58**
		LdH	**−48.4 ± 4.3**	**−45.6 ± 5.6 ^a^**	**−54.3 ± 3.8 ^a^**	**0.02**	**0.41**
	Max HL retraction time (% jump stride)	TrH	70.2 ± 2.6	72.6 ± 2.6	70.6 ± 1.5	0.25	0.21
		LdH	71.3 ± 1.3	72.2 ± 3.1	68.5 ± 3.9	0.16	0.25
Impulsion	HL A1 stance duration (s)	TrH	0.2 ± 0.0	0.2 ± 0.0	0.2 ± 0.0	0.07	0.41
		LdH	**0.2 ± 0.0 ^a^**	**0.2 ± 0.0**	**0.3 ± 0.0 ^a^**	**0.04**	**0.42**
	FL A1 stance duration (s)	TrF	**0.2 ± 0.0 ^a^**	**0.2 ± 0.0 ^b^**	**0.3 ± 0.0 ^a,b^**	**0.01**	**0.51**
		LdF	**0.2 ± 0.0 ^a^**	**0.2 ± 0.0**	**0.3 ± 0.0 ^a^**	**0.02**	**0.44**
	HL jump stance duration (s)	TrH	0.2 ± 0.0	0.2 ± 0.0	0.3 ± 0.0	0.11	0.29
		LdH	0.2 ± 0.0	0.2 ± 0.0	0.3 ± 0.0	0.07	0.33
	Duty factor (% A1 stride)	TrH *	52.1 ± 4.6	58.4 ± 4.7	57.3 ± 2.3	0.10	0.37
		LdH	55.3 ± 7.2	59.1 ± 2.4	59.5 ± 1.9	0.35	0.17
	Duty factor (% jump stride)	TrH	**27.7 ± 4.8 ^a^**	**31.5 ± 3.8**	**36.7 ± 4.3 ^a^**	**0.02**	**0.47**
		LdH	**29.5 ± 4.9 ^a^**	**32.2 ± 3.2**	**37.6 ± 3.5 ^a^**	**0.02**	**0.47**
	Z_CM_ (m)	TrH	**0.5 ± 0.0 ^a,b^**	**0.4 ± 0.0 ^a,c^**	**0.3 ± 0.1 ^b,c^**	**0.00**	**0.78**
		LdH	**0.5 ± 0.0 ^a,b^**	**0.4 ± 0.1 ^a,c^**	**0.3 ± 0.0 ^b,c^**	**0.00**	**0.81**
	Z_CM_ time (% jump stride)	TrH	46.4 ± 1.7	47.5 ± 2.1	44.9 ± 3.3	0.25	0.21
		LdH	47.5 ± 2.2	46.9 ± 3.8	45.0 ± 5.1	0.57	0.08
	${\dot{Z}}_{CM}$ (m/s)	TrH	**2.1 ± 0.2 ^a^**	**1.8 ± 0.3**	**1.4 ± 0.3 ^a^**	**0.00**	**0.57**
		LdH	**2.2 ± 0.1 ^a^**	**1.8 ± 0.3 ^b^**	**1.4 ± 0.3 ^a,b^**	**0.00**	**0.62**
	${\dot{Z}}_{CM}$ time (% jump stride)	TrH	**19.1 ± 1.9 ^a^**	**22.3 ± 4.3**	**25.2 ± 1.0 ^a^**	**0.03**	**0.43**
		LdH	21.0 ± 3.0	22.5 ± 2.4	23.9 ± 3.9	0.43	0.13
	${\ddot{Z}}_{CM}$ (m/s^2^)	TrH	17.0 ± 6.6	17.8 ± 6.2	14.6 ± 2.7	0.61	0.07
		LdH	16.8 ± 3.5	15.9 ± 4.0	12.7 ± 2.1	0.16	0.23
	${\ddot{Z}}_{CM}$ time (% jump stride)	TrH	**8.0 ± 1.6^a^**	**11.1 ± 3.2**	**14.7 ± 2.4^a^**	**0.01**	**0.55**
		LdH	9.9 ± 1.7	11.4 ± 4.2	13.7 ± 3.8	0.37	0.15
	A1 stride vel (m/s)	TrH	**6.5 ± 0.6^a^**	**6.1 ± 0.4**	**5.5 ± 0.5^a^**	**0.04**	**0.49**
		LdH	6.6 ± 0.5	6.2 ± 0.3	5.8 ± 0.6	0.05	0.37
	Jump stride vel (m/s)	TrH *	**6.7 ± 0.7**	**6.3 ± 0.3**	**5.7 ± 0.7**	**0.04**	**0.41**
		LdH *	6.6 ± 0.2	6.5 ± 0.3	5.9 ± 0.7	0.10	0.30
Engagement	Max hock flex take-off (°)	TrH	31.2 ± 2.0	33.2 ± 7.1	32.2 ± 6.4	0.87	0.02
		LdH	26.5 ± 6.4	33.5 ± 7.8	29.4 ± 7.7	0.30	0.16
	Max hock flex take-off time (% jump stride)	TrH	**11.8 ± 1.1 ^a^**	**14.1 ± 3.1**	**16.1 ± 1.0 ^a^**	**0.04**	**0.42**
		LdH	12.3 ± 0.7	14.4 ± 3.0	16.6 ± 2.8	0.08	0.32
	Max stifle flex take-off (°)	TrH *	28.6 ± 3.9	31.5 ± 5.9	34.1 ± 6.1	0.37	0.14
		LdH *	28.0 ± 5.4	28.7 ± 6.0	29.1 ± 8.9	0.97	0.01
	Max stifle flex take-off time (% jump stride)	TrH	**14.6 ± 0.9 ^a^**	**17.5 ± 3.0**	**19.8 ± 1.7 ^a^**	**0.02**	**0.50**
		LdH	**15.3 ± 1.7 ^a^**	**18.3 ± 2.3**	**19.8 ± 2.4 ^a^**	**0.03**	**0.43**
	Max hip flex take-off (°)	TrH	−2.9 ± 1.3	−5.2 ± 3.0	−5.1 ± 1.8	0.29	0.17
		LdH	−2.1 ± 4.2	−3.3 ± 1.9	−4.0 ± 2.2	0.57	0.08
	Max hip flex take-off time (% jump stride)	TrH	11.2 ± 1.8	13.0 ± 2.8	12.3 ± 1.3	0.44	0.13
		LdH	12.3 ± 3.3	13.1 ± 1.8	14.0 ± 2.0	0.57	0.08
	Max HL shortening take-off (m)	TrH	−0.2 ± 0.0	−0.1 ± 0.0	−0.1 ± 0.0	0.08	0.32
		LdH	−0.2 ± 0.0	−0.1 ± 0.0	−0.1 ± 0.0	0.75	0.04
	Max HL shortening take-off time (% jump stride)	TrH	**11.5 ± 0.6 ^a^**	**14.5 ± 2.8**	**16.5 ± 1.5 ^a^**	**0.01**	**0.54**
		LdH	12.3 ± 0.9	15.1 ± 2.2	16.2 ± 1.8	0.05	0.42
	Max HL protraction A1 stride (°)	TrH	22.2 ± 1.5	21.0 ± 3.4	20.5 ± 3.7	0.72	0.05
		LdH	20.1 ± 2.5	20.0 ± 6.8	22.4 ± 4.2	0.68	0.06

* Between-limb differences are significant at the 0.05 level. Abbreviations: Hindlimb (HL), forelimb (FL), leading hindlimb (LdH), trailing hindlimb (TrH), leading forelimb (LdF), trailing forelimb (TrF), approach stride (A1 stride), CM vertical displacement ($Z_{CM}$), CM vertical velocity (${\dot{Z}}_{CM}$), CM vertical acceleration (${\ddot{Z}}_{CM})$, and flexion (flex).

**Table 2:** Descriptive statistics (mean ± standard deviation) for sEMG variables within each subgroup and muscle. Between-group differences are presented for each variable as p-values and effect sizes (eta^2^). Within each row, significant main effects (*p* < 0.05) for between-group differences are denoted by bold text and significant differences (*p* < 0.05) between groups are represented by corresponding superscripts (^a–b^). Significant differences (*p* < 0.05) between groups are denoted by bold text and between limbs as grey shaded cells.

Muscle	Stride	sEMG Variable (% stride)	Limb	Group			*p*	eta^2^
				High_CM_	Int._CM_	Low_CM_		
Middle gluteal	A1	Peak amplitude	TrH	8.1 ± 11.5	19.4 ± 19.5	20.5 ± 28.6	0.65	0.10
			LdH	11.5 ± 6.5	16.7 ± 19.2	17.7 ± 17.2	0.78	0.05
		Activity duration	TrH	55.4 ± 15.2	78.0 ± 14.5	70.0 ± 17.2	0.17	0.35
			LdH	66.6 ± 16.3	80.3 ± 11.6	87.6 ± 18.8	0.20	0.30
		A1 activity offset	TrH	**35.4 ± 3.8 ^a,b^**	**57.9 ± 13.4 ^a^**	**61.8 ± 2.5 ^b^**	**0.02**	**0.70**
			LdH	44.9 ± 15.2	51.1 ± 20.4	55.8 ± 3.0	0.70	0.10
		Take-off activity onset	TrH	81.4 ± 4.7	79.9 ± 4.9	71.2 ± 25.1	0.59	0.13
			LdH	79.5 ± 4.3	76.5 ± 9.2	83.0 ± 20.8	0.77	0.07
	Jump	Peak amplitude	TrH	6.7 ± 5.1	7.0 ± 1.9	9.2 ± 4.2	0.63	0.10
			LdH	8.5 ± 2.5	10.6 ± 7.2	11.6 ± 2.2	0.67	0.76
		Activity duration	TrH	**31.1 ± 9.4 ^a^**	**46.2 ± 11.8**	**61.6 ± 16.9 ^a^**	**0.03**	**0.55**
			LdH	**28.1 ± 6.9 ^a^**	**43.3 ± 14.6**	**67.4 ± 18.4 ^a^**	**0.01**	**0.61**
		Take-off activity offset	TrH	24.3 ± 7.3	25.9 ±9.5	34.9 ± 17.3	0.44	0.17
			LdH	20.2 ± 1.7	26.8 ±7.6	39.2 ± 17.2	0.08	0.40
		Landing activity onset	TrH	**91.6 ± 3.0 ^a^**	**82.8 ± 7.8**	**76.9 ± 3.4 ^a^**	**0.02**	**0.61**
			LdH	**89.5 ± 3.7 ^a^**	**87.9 ± 6.0**	**77.6 ± 5.1 ^a^**	**0.03**	**0.56**
Biceps femoris	A1	Peak amplitude	TrH	16.8 ± 3.8	33.2 ± 13.2	16.7 ± 1.5	0.07	0.53
			LdH	23.4 ± 4.2	35.3 ± 16.9	24.5 ± 11.4	0.30	0.22
		Activity duration	TrH	73.2 ± 15.6	73.6 ± 10.3	71.4 ± 5.6	0.98	0.01
			LdH	70.3 ± 7.7	81.5 ± 10.0	78.5 ± 14.8	0.26	0.24
		A1 activity offset	TrH *	**40.0 ± 4.0 ^a^**	**58.0 ± 10.4 ^a^**	**40.7 ± 4.7**	**0.02**	**0.66**
			LdH *	45.4 ± 3.5	60.2 ± 16.5	49.6 ± 11.4	0.18	0.29
		Take-off activity onset	TrH	72.5 ± 16.3	84.4 ± 7.7	69.3 ± 0.9	0.29	0.30
			LdH	75.2 ± 10.8	78.6 ± 10.6	71.1 ± 7.4	0.62	0.09
	Jump	Peak amplitude	TrH	9.5 ± 3.0	8.0 ± 3.8	12.1 ± 0.5	0.24	0.27
			LdH	9.4 ± 3.3	10.8 ± 7.3	11.5 ± 1.9	0.86	0.03
		Activity duration	TrH	36.9 ± 10.9	41.6 ± 11.3	47.9 ± 9.9	0.45	0.16
			LdH	40.9 ± 15.8	45.7 ± 12.9	39.2 ± 16.7	0.79	0.05
		Take-off activity offset	TrH *	31.0 ± 9.6	35.7 ± 12.0	36.4 ± 15.5	0.80	0.05
			LdH *	37.0 ± 13.0	41.6 ± 11.8	35.5 ± 20.1	0.80	0.04
		Landing activity onset	TrH	94.1 ± 4.6	94.6 ± 3.5	88.8 ± 6.3	0.24	0.27
			LdH	97.2 ± 6.2	97.1 ± 7.5	97.1 ± 6.0	1.00	0.00
Triceps brachii	A1	Peak amplitude	TrF	49.6 ± 23.7	56.7 ± 28.8	40.1 ± 21.4	0.78	0.09
			LdF	56.7 ± 14.2	63.5 ± 8.1	62.5 ± 34.0	0.83	0.04
		Activity duration	TrF	91.2 ± 15.2	71.2 ± 20.7	87.01 ± 0.9	0.37	0.33
			LdF	90.3 ± 13.3	65.4 ± 21.0	93.6 ± 5.5	0.05	0.46
		A1 activity offset	TrF	55.8 ± 3.7	59.4 ± 13.1	71.0 ± 22.6	0.72	0.28
			LdF	74.7	65.9 ± 8.9	76.8 ± 0.5	0.36	0.50
		Take-off activity onset	TrF	76.5 ± 1.7	79.0 ± 1.9	66.01	0.06	0.94
			LdF		73.9 ± 17.6	86.3 ± 0.6	0.40	0.18
	Jump	Peak amplitude	TrF	71.8 ± 19.3	75.3 ± 8.7	73.4 ± 8.0	0.94	0.02
			LdF	61.0 ± 44.1	56.0 ± 34.4	70.5 ± 4.8	0.83	0.04
		Activity duration	TrF	54.9 ± 22.7	55.3 ± 7.8	70.2 ± 0.9	0.31	0.28
			LdF	45.2 ± 9.4	62.7 ± 16.9	60.6 ± 12.8	0.26	0.25
		FL take-off activity offset	TrF	21.3 ± 4.6	14.2 ± 8.8	21.3 ± 9.0	0.44	0.24
			LdF	15.4 ± 11.3	18.8 ± 11.1	11.9 ± 3.8	0.65	0.10
		FL landing activity onset	TrF	61.5 ± 10.9	58.1 ± 2.0	48.1 ± 19.0	0.40	0.23
			LdF	66.1 ± 5.3	58.0 ± 4.0	58.0 ± 10.3	0.16	0.37

* Between-limb differences are significant at the 0.05 level. Abbreviations: forelimb (FL), leading hindlimb (LdH), trailing hindlimb (TrH), leading forelimb (LdF), trailing forelimb (TrF), and approach stride (A1 stride).

**Table 3:** Correlations between kinematic and sEMG activity timing variables where significant (*p* < 0.05) between-group differences were observed. Pearson correlation coefficients (r) are presented for each comparison. Significant (*p* < 0.05) correlations are denoted by bold text.

Kinematic Variable	Limb	Gluteal A1 Offset		Gluteal Jump Duration		Gluteal Jump Landing Onset		Biceps Femoris A1 Offset	
		TrH	LdH	TrH	LdH	TrH	LdH	TrH	LdH
Max stifle flex take-off time (% jump stride)	TrH	0.29	−0.03	0.47	0.45	−0.47	−0.50	−0.38	−0.44
	LdH	0.15	0.01	0.58	0.60 *	−0.37	−0.42	−0.02	0.02
Max HL retraction (°)	TrH	0.02	0.16	−0.22	−0.31	0.02	0.52	0.79 **	0.45
	LdH	−0.15	0.10	−0.36	−0.50	0.13	0.52	0.83 **	0.37
Z_CM_ (m)	TrH	−0.56	−0.17	−0.71 *	−0.65 *	0.65 *	0.67 *	0.10	0.12
	LdH	−0.63	−0.11	−0.76 **	−0.78 **	0.78 **	0.75 **	0.13	0.08
${\dot{Z}}_{CM}$ (m/s)	TrH	−0.41	0.15	−0.64 *	−0.60 *	0.67 *	0.57	0.18	0.27
	LdH	−0.48	−0.03	−0.65 *	−0.65 *	0.65 *	0.60 *	0.25	0.21
${\dot{Z}}_{CM}$ time (% jump stride)	TrH	0.12	0.08	0.25	0.23	−0.43	−0.28	−0.20	−0.38
	LdH	−0.11	0.08	−0.07	−0.04	0.05	0.09	−0.04	−0.03
${\ddot{Z}}_{CM}$ time (% jump stride)	TrH	0.22	0.14	0.48	0.43	−0.47	−0.36	−0.29	−0.36
	LdH	−0.19	−0.36	−0.07	−0.11	0.11	0.06	−0.25	−0.33
Max hock flex take-off time (% jump stride)	TrH	0.16	−0.23	0.42	0.43	−0.50	−0.43	−0.33	−0.44
	LdH	0.01	0.04	0.31	0.38	−0.11	−0.40	−0.12	−0.12
Max HL shortening time (% jump stride)	TrH	0.24	0.36	0.60 *	0.62 *	0.01	−0.66 *	−0.34	−0.01
	LdH	0.53	0.25	0.58	0.62 *	−0.03	−0.54	0.06	0.27
Max HL shortening take-off time (% jump stride)	TrH	0.30	0.04	0.41	0.41	−0.47	−0.44	−0.24	−0.39
	LdH	0.15	0.03	0.34	0.36	−0.35	−0.21	−0.06	−0.18
HL A1 stance (s)	TrH	0.58	0.53	0.40	0.43	−0.61	−0.47	−0.13	−0.07
	LdH	0.62	0.53	0.36	0.35	−0.78 *	−0.32	−0.02	−0.01
A1 stride velocity (m/s)	TrH	0.55	0.43	0.52	0.72 *	−0.82 *	−0.61	−0.16	−0.15
	LdH	0.46	0.11	0.24	0.43	−0.61	−0.22	−0.19	−0.14
Duty factor (% jump stride)	TrH	0.24	−0.05	0.39	0.54	−0.69 *	−0.46	−0.23	−0.27
	LdH	0.07	−0.14	0.32	0.58 *	−0.48	−0.44	−0.26	−0.10

** Correlation is significant at the 0.01 level (two-tailed). * Correlation is significant at the 0.05 level (two tailed). Abbreviations: Hindlimb (HL), leading hindlimb (LdH), trailing hindlimb (TrH), approach stride (A1 stride), CM vertical displacement ($Z_{CM}$), CM vertical velocity (${\dot{Z}}_{CM}$), CM vertical acceleration (${\ddot{Z}}_{CM}$), and flexion (flex).

## Data Availability

The data presented in this study are openly available in UCLanData at https://doi.org/10.17030/uclan.data.00000288, reference number 288.

## Supplementary Materials

The following are available online at https://www.mdpi.com/2076-2615/11/2/414/s1, Figure S1: Mean and standard deviation time–angle curves (°) for kinematic variables within the joint articulation theme; Figure S2: Mean and standard deviation time–angle curves (°) for kinematic variables within the engagement theme; Figure S3: SPM results for gluteal sEMG waveforms; Figure S4: SPM results for biceps femoris sEMG waveforms; Figure S5: SPM results for triceps sEMG waveforms; Table S1: Kinematic measurement techniques for equestrian-derived performance indicators. Table S2: Pairwise comparisons for kinematic variables where a significant main effect was found between groups; Table S3: Pairwise comparisons for sEMG activity timing variables where a significant main effect was found between groups; Table S4: Correlations between kinematic variables were significant between-group differences were observed and the discriminative performance indicator, Z_CM_.

## References

[1] Fédération Equestre Internationale Main Jumping. https://inside.fei.org/fei/disc/jumping.

[2] Federation Equestre Internationale FEI World of Sport-FEI Annual Report 2019. https://inside.fei.org/fei/about-fei/publications/fei-annual-report/2019/feiworldofsport/.

[3] Górecka-Bruzda A., Chruszczewski M.H., Jaworski Z., Golonka M., Jezierski T., Długosz B., Pieszka M. (2011). Looking for an ideal horse: Rider preferences. Anthrozoös.

[4] Fédération Equestre Internationale Jumping Rules. https://inside.fei.org/sites/default/files/Jumping_Rules_2020_clean.pdf.

[5] Cassiat G., Pourcelot P., Tavernier L., Geiger D., Denoix J.M., Degueurce D. (2004). Influence of individual competition level on back kinematics of horses jumping a vertical fence. Equine Vet. J..

[6] Bobbert M.F., Santamaría S., van Weeren P.R., Back W., Barneveld A. (2005). Can jumping capacity of adult show jumping horses be predicted on the basis of submaximal free jumps at foal age? A longitudinal study. Vet. J..

[7] Santamaría S., Bobbert M.F., Back W., Barneveld A., van Weeren P.R. (2005). Effect of early training on the jumping technique of horses. Am. J. Vet. Res..

[8] Santamaría S., Back W., Van Weeren P., Knaap J., Barneveld A. (2002). Jumping characteristics of naive foals: Lead changes and description of temporal and linear parameters. Equine Vet. J..

[9] Clayton H.M. (1996). Time-motion analysis of show jumping competitions. J. Equine Vet. Sci..

[10] Smith R., Birch H., Patterson-Kane J., Firth E., Williams L., Cherdchutham W., Van Weeren P.R., Goodship A. (1999). Should equine athletes commence training during skeletal development?: Changes in tendon matrix associated with development, ageing, function and exercise. Equine Vet. J..

[11] St George L., Hobbs S.J., Sinclair J., Richards J., Roddam H. (2019). Does equestrian knowledge and experience influence selection and training practices for showjumping horses?. Comp. Exerc. Physiol..

[12] Williams J. (2013). Performance analysis in equestrian sport. Comp. Exerc. Physiol..

[13] Hughes M.D., Bartlett R.M. (2002). The use of performance indicators in performance analysis. J. Sports Sci..

[14] Santamaría S., Bobbert M.F., Back W., Barneveld A., van Weeren P.R. (2004). Evaluation of consistency of jumping technique in horses between the ages of 6 months and 4 years. Am. J. Vet. Res..

[15] Van den Bogert A., Jansen M.O., Deuel N.R. (1994). Kinematics of the hind limb push-off in elite show jumping horses. Equine Vet. J..

[16] Powers P., Harrison A. (2000). A study on the techniques used by untrained horses during loose jumping. J. Equine Vet. Sci..

[17] Schambardt H., Merkens H., Vogel V., Willekens C. (1993). External loads on the limbs of jumping horses at take-off and landing. Am. J. Vet. Res..

[18] Colborne G., Clayton H.M., Lanovaz J. (1995). Factors that influence vertical velocity during take off over a water jump. Equine Vet. J..

[19] Powers P. (2005). Equestrian: Linear kinematics at take-off in horses jumping the wall in an international Puissance competition. Sports Biomech..

[20] Lewczuk D., Wejer J., Sobieraj D. (2007). Analysis of angles of taking off, landing, and work of limbs in horses jumping above the spread obstacle of different structure. Anim. Sci. Pap. Rep..

[21] Barrey E., Blanchard G., Orange F. Influence of the level of competition, breed, sex, and genetic factors on stride kinematics of show jumping horses. Proceedings of the 12th Meeting of the Association for Equine Sports Medicine.

[22] Clayton H.M., Barlow D.A. (1989). The effect of fence height and width on the limb placements of show jumping horses. J. Equine Vet. Sci..

[23] Clayton H.M., Colborne G.R., Burns T.E. (1995). Kinematic analysis of successful and unsuccessful attempts to clear a water jump. Equine Vet. J..

[24] Lewczuk D. (2013). Effect of the judge and definition of the trait for horse free jumping evaluation. Arch. Anim. Breed..

[25] Barrey E., Galloux P. (1997). Analysis of the equine jumping technique by accelerometry. Equine Vet. J..

[26] Santamaría S., Bobbert M.F., Back W., Barneveld A., van Weeren P.R. (2004). Variation in free jumping technique within and among horses with little experience in show jumping. Am. J. Vet. Res..

[27] Galloux P., Barrey E. (1997). Components of the total kinetic moment in jumping horses. Equine Vet. J..

[28] Harrison S.M., Whitton R.C., King M., Haussler K.K., Kawcak C.E., Stover S.M., Pandy M.G. (2012). Forelimb muscle activity during equine locomotion. J. Exp. Biol..

[29] Jansen M., van Raaij J., Van den Bogert A., Schamhardt H., Hartman W. (1992). Quantitative analysis of computer-averaged electromyographic profiles of intrinsic limb muscles in ponies at the walk. Am. J. Vet. Res..

[30] Zsoldos R., Kotschwar A., Kotschwar A., Groesel M., Licka T., Peham C. (2010). Electromyography activity of the equine splenius muscle and neck kinematics during walk and trot on the treadmill. Equine Vet. J..

[31] Zsoldos R., Kotschwar A., Kotschwar A., Rodriguez C., Peham C., Licka T. (2010). Activity of the equine rectus abdominis and oblique external abdominal muscles measured by surface EMG during walk and trot on the treadmill. Equine Vet. J..

[32] St George L., Roy S., Richards J., Sinclair J., Hobbs S.J. (2019). Surface EMG signal normalisation and filtering improves sensitivity of equine gait analysis. Comp. Exerc. Physiol..

[33] Robert C., Valette J., Degueurce C., Denoix J. (1999). Correlation between surface electromyography and kinematics of the hindlimb of horses at trot on a treadmill. Cells Tissues Organs.

[34] Licka T., Frey A., Peham C. (2009). Electromyographic activity of the longissimus dorsi muscles in horses when walking on a treadmill. Vet. J..

[35] Licka T.F., Peham C., Frey A. (2004). Electromyographic activity of the longissimus dorsi muscles in horses during trotting on a treadmill. Am. J. Vet. Res..

[36] St George L., Williams J. (2013). Electromyographic evaluation of approach stride, jump stride and intermediate stride in selected superficial muscles of the jumping horse: A preliminary study. Comp. Exerc. Physiol..

[37] Giovagnoli C., Pieramati G., Castellano G., Reitano M., Silvestrelli M. Analysis of neck muscle (Splenius) activity during jumping by surface video-electromyography technique. Proceedings of the Conference on Equine Sports Medicine and Science (CESMAS).

[38] St George L., Hobbs S.J., Richards J., Sinclair J., Holt D., Roy S. (2018). The effect of cut-off frequency when high-pass filtering equine sEMG signals during locomotion. J. Electromyogr. Kinesiol..

[39] Bobbert M.F., Santamaría S. (2005). Contribution of the forelimbs and hindlimbs of the horse to mechanical energy changes in jumping. J. Exp. Biol..

[40] Winfield J. (2010). What industry requires from the application of research from equine science. Adv. Anim. Biosci..

[41] McGarry T. (2009). Applied and theoretical perspectives of performance analysis in sport: Scientific issues and challenges. Int. J. Perform. Anal..

[42] Robert C., Audigié F., Valette J., Pourcelot P., Denoix J.M. (2001). Effects of treadmill speed on the mechanics of the back in the trotting saddlehorse. Equine Vet. J..

[43] Hodson-Tole E. (2006). Effects of treadmill inclination and speed on forelimb muscle activity and kinematics in the horse. Equine Comp. Exerc. Physiol..

[44] Schuurman S.O., Kersten W., Weijs W.A. (2003). The equine hind limb is actively stabilized during standing. J. Anat..

[45] Zaneb H., Kaufmann V., Licka T., Peham C., Stanek C. Determination of position of surface electromyographic electrodes for selected equine muscles. Proceedings of the Noraxon EMG Meeting 2007.

[46] Cram J.R., Rommen D. (1989). Effects of skin preparation on data collected using an EMG muscle-scanning procedure. Biofeedback Self-Regul..

[47] Clancy E.A., Morin E.L., Merletti R. (2002). Sampling, noise-reduction and amplitude estimation issues in surface electromyography. J. Electromyogr. Kinesiol..

[48] Hermens H.J., Freriks B., Disselhorst-Klug C., Rau G. (2000). Development of recommendations for SEMG sensors and sensor placement procedures. J. Electromyogr. Kinesiol..

[49] De Luca C.J. (1997). The use of surface electromyography in biomechanics. J. Appl. Biomech..

[50] Clayton H.M., can Weeren P.R., Back W., Clayton H.M. (2013). Performance in Equestrian Sports. Equine Locomotion.

[51] Hobbs S.J., Richards J., Clayton H.M. (2014). The effect of centre of mass location on sagittal plane moments around the centre of mass in trotting horses. J. Biomech..

[52] Holt D., St George L., Clayton H., Hobbs S.J. (2017). A simple method for equine kinematic gait event detection. Equine Vet. J..

[53] Clayton H.M. (1989). Terminology for the description of equine jumping kinematics. J. Equine Vet. Sci..

[54] FEI (2007). Dressage Handbook-Guidelines for Judging.

[55] Biewener A.A. (1983). Allometry of quadrupedal locomotion: The scaling of duty factor, bone curvature and limb orientation to body size. J. Exp. Biol..

[56] Back W., Schamhardt H., Savelberg H., Van den Bogert A., Bruin G., Hartman W., Barneveld A. (1995). How the horse moves: 1. Significance of graphical representations of equine forelimb kinematics. Equine Vet. J..

[57] Back W., Schamhardt H., Savelberg H., Van Den Bogert A., Bruin G., Hartman W., Barneveld A. (1995). How the horse moves: 2. Significance of graphical representations of equine hind limb kinematics. Equine Vet. J..

[58] Bonato P., D’Alessio T., Knaflitz M.A. (1998). Statistical method for the measurement of muscle activation intervals from surface myoelectric signal during gait. IEEE Trans. Biomed. Eng..

[59] Merlo A., Farina D., Merletti R. (2003). A fast and reliable technique for muscle activity detection from surface EMG signals. IEEE trans. Biomed. Eng..

[60] Bullock-Saxton J.E. (1994). Local sensation changes and altered hip muscle function following severe ankle sprain. Phys. Ther..

[61] Bogey R.A., Barnes L.A., Perry J. (1992). Computer algorithms to characterize individual subject EMG profiles during gait. Arch. Phys. Med Rehabil..

[62] Clayton H., Barlow D. (1991). Stride characteristics of four Grand Prix jumping horses. Equine Exerc. Phys..

[63] Patil S., Dixon J., White L.C., Jones A.P., Hui A.C. (2011). An electromyographic exploratory study comparing the difference in the onset of hamstring and quadriceps contraction in patients with anterior knee pain. Knee.

[64] Vera M.J., Dubravka B., Nikola J., Vojin I., Bojana P.-B. (2013). Detecting and removing outlier (s) in electromyographic gait-related patterns. J. Appl. Stat..

[65] Stålberg E., Bischoff C., Falck B. (1994). Outliers, a way to detect abnormality in quantitative EMG. Muscle Nerve.

[66] Dutto D.J., Hoyt D.F., Clayton H.M., Cogger E.A., Wickler S.J. (2004). Moments and power generated by the horse (Equus caballus) hind limb during jumping. J. Exp. Biol..

[67] Powers P., Harrison A. (1999). Models for biomechanical analysis of jumping horses. J. Equine Vet. Sci..

[68] Witte T., Hirst C., Wilson A. (2006). Effect of speed on stride parameters in racehorses at gallop in field conditions. J. Exp. Biol..

[69] Witte T., Knill K., Wilson A. (2004). Determination of peak vertical ground reaction force from duty factor in the horse (Equus caballus). J. Exp. Biol..

[70] Payne R., Hutchinson J., Robilliard J., Smith N., Wilson A. (2005). Functional specialisation of pelvic limb anatomy in horses (Equus caballus). J. Anat..

[71] Denoix J.M. (2014). Biomechanics and Physical Training of the Horse.

[72] Komi P.V., Linnamo V., Silventoinen P., Sillanpää M. (2000). Force and EMG power spectrum during eccentric and concentric actions. Med. Sci. Sports Exerc..

[73] Santamaría S., Bobbert M.F., Back W., Barneveld A., van Weeren P.R. (2006). Can early training of show jumpers bias outcome of selection events?. Livest. Sci..

[74] Watson J., Wilson A. (2007). Muscle architecture of biceps brachii, triceps brachii and supraspinatus in the horse. J. Anat..

[75] Wilson A.M., McGuigan M.P., Su A., van den Bogert A.J. (2001). Horses damp the spring in their step. Nature.

[76] Hobbs S.J., Robinson M.A., Clayton H.M. (2018). A simple method of equine limb force vector analysis and its potential applications. PeerJ.

[77] Halaki M., Ginn K. (2012). Normalization of EMG signals: To normalize or not to normalize and what to normalize to. Computational Intelligence in Electromyography Analysis-A Perspective on Current Applications and Future Challenges.

[78] Lehman G.J., McGill S.M. (1999). The importance of normalization in the interpretation of surface electromyography: A proof of principle. J. Manip. Physiol. Ther..

[79] Hof A., Elzinga H., Grimmius W., Halbertsma J. (2002). Speed dependence of averaged EMG profiles in walking. Gait Posture.

[80] Byström A., Clayton H., Hernlund E., Rhodin M., Egenvall A. (2020). Equestrian and biomechanical perspectives on laterality in the horse. Comp. Exerc. Physiol..

[81] Hodges P.W., Bui B.H. (1996). A comparison of computer-based methods for the determination of onset of muscle contraction using electromyography. Electroencephalogr. Clin. Neurophysiol..

[82] Özgünen K.T., Çelik U., Kurdak S.S. (2010). Determination of an optimal threshold value for muscle activity detection in EMG analysis. J. Sports Sci. Med..

[83] Micera S., Sabatini A.M., Dario P. (1998). An algorithm for detecting the onset of muscle contraction by EMG signal processing. Med. Eng. Phys..

