# Supplementary material for: Muscle Function and Kinematics during Submaximal Equine Jumping: What Can Objective Outcomes Tell Us about Athletic Performance Indicators?
Source: Animals (Basel). 2021 Feb 5;11(2):414. doi: 10.3390/ani11020414 (PMC7915507; doi:10.3390/ani11020414)
Supplement: Supplementary file 1 [file animals-11-00414-s001.zip › St George et al Supplementary Info/Supplementary Info.docx]

**Table S1:** Kinematic measurement techniques for equestrian-derived performance indicators. All variables are calculated within the jump stride unless otherwise stated. For outcome measures where an additional spatiotemporal measure is listed (% stride), the timing of each described outcome measure is calculated and normalised to jump stride duration.

| Theme | Jump performance trait | Kinematic outcome measure | Kinematic Measurement Technique |
| --- | --- | --- | --- |
| Joint articulation | Ability to “use” and elevate the shoulder and to “back off” fence | Max shoulder flex (**°**) (% jump stride) | Maximum value from normalized shoulder joint-angle curve. |
|  |  | Max scapula angle (**°**)  (% jump stride) | Maximum value from scapula segment time-angle curve plotted within LCS (Y axis). |
|  | Ability to “tuck up”, elevate, and lift FL at take-off | Max FL shortening (m)  (% jump stride) | Minimum value from FL segment length-time signal, calculated by subtracting proximal (lateral epicondyle of humerus) from distal (fore DIPJ) vertical coordinates and normalizing to standing segment length. |
|  |  | Max carpus, elbow flex (**°**) (% jump stride) | Maximum value from normalized carpus and elbow joint-angle curves. |
|  |  | Max radius angle (**°**) (% jump stride) | Maximum value from radius segment time-angle curve plotted within LCS (Y axis). |
|  | Ability to “tuck up” the HL. | Max HL shortening (m)  (% jump stride) | As above for “Max FL shortening”, using proximal (tubera coxae) and distal (hind DIPJ) ends of the HL segment. |
|  | Ability to “open out behind”, “throw away the HL” | Max HL retraction (**°**) (% jump stride) | Minimum value from HL segment time-angle curve plotted within LCS (Y axis). |
| Impulsion | Power, strength, ability to push-off and pat/stamp the ground [take-off] | HL stance duration (s) | Time between HL hoof impact and lift-off events measured at jump and A1 strides. |
|  |  | Duty factor (% stride) | Temporal proportion of stride duration where HL is in contact with the ground (% stance) (Biewener, 1983). Measured at jump and A1 strides. |
|  |  | Z**_CM_** (m)  (% jump stride) | Maximum vertical coordinate of CM marker within the LCS (z-axis). Normalized to standing CM height. |
|  |  | ${\dot{\boldsymbol{Z}}}_{\boldsymbol{CM}}$ (m/s)  (% jump stride) | Maximum vertical velocity of CM, calculated as first derivative of CM marker coordinates within the LCS (z-axis). |
|  |  | ${\ddot{\boldsymbol{Z}}}_{\boldsymbol{CM}}$ (m/s^2^)  (% jump stride) | Maximum vertical acceleration of CM during HL stance phase at take-off, calculated as second derivative of CM marker coordinates within the LCS (z-axis). |
|  | Forward | Stride velocity (m/s) | First derivative of croup (tubera sacrale) marker coordinates within the LCS (y-axis) averaged over A1 and jump strides. |
|  | Quick, snappy FL lift-off [take-off] | FL A1 stance duration (s) | Time between FL hoof impact and lift-off events measured at A1 stride. |
| Engaged | Ability to “take weight behind”, “sit” and “rock onto hocks” [take off] | Max hock, stifle, hip joint flex take-off (**°**) (% jump stride) | Maximum value from normalized hock, stifle and hip joint-angle curves during HL stance phase at take-off. |
|  |  | Max HL shortening take-off (% jump stride) | As above for “Max HL shortening”, using minimum value during HL stance phase at take-off. |
|  | Ability to bring the HL underneath body | Max HL protraction A1 stride (°) | Maximum value from HL segment time-angle curve plotted within LCS (Y axis) during A1 stride. |

**Table S2.** Pairwise comparisons for kinematic variables where a significant main effect was found between groups. Between group differences are presented for each variable as mean difference (MD), P-values and 95% confidence intervals (95% CI). Significant differences between groups are denoted by bold text.

| Theme | Kinematic Variable | Limb |  | High_CM_ – Int._CM_ | High_CM_ – Low_CM_ | Int_CM_. – Low_CM_ |
| --- | --- | --- | --- | --- | --- | --- |
| Joint articulation | Max scapula angle time (% jump stride) | TrF | MD | -4.1 | -4.6 | -0.5 |
|  |  |  | P value | 0.06 | **0.04** | 1.00 |
|  |  |  | 95% CI | -8.3, 0.2 | -9.0, -0.1 | -4.5, 3.5 |
|  | Max radius angle (**°**) | LdF | MD | -6.9 | 3.4 | 10.4 |
|  |  |  | P value | 0.27 | 1.00 | **0.05** |
|  |  |  | 95% CI | -17.2, 3.4 | -7.7, 14.6 | 0.1, 20.7 |
|  | Max HL shortening time  (% jump stride) | LdH | MD | -11.9 | -15.2 | -3.3 |
|  |  |  | P value | 0.07 | **0.03** | 1.00 |
|  |  |  | 95% CI | -24.7, 0.9 | -28.8, -1.5 | -15.2, 8.7 |
|  | Max HL retraction (°) | TrH | MD | -4.2 | 2.3 | 6.4 |
|  |  |  | P value | 0.08 | 0.68 | **0.00** |
|  |  |  | 95% CI | -8.8, 0.4 | -2.6, 7.2 | 2.1, 10.7 |
|  |  | LdH | MD | -2.8 | 6.0 | 8.8 |
|  |  |  | P value | 1.00 | 0.21 | **0.02** |
|  |  |  | 95% CI | -10.4, 4.8 | -2.2, 14.2 | 1.2, 16.4 |
| Impulsion | HL A1 stance duration (s) | LdH | MD | -0.03 | -0.06 | -0.03 |
|  |  |  | P value | 0.46 | **0.04** | 0.53 |
|  |  |  | 95% CI | -0.09, 0.02 | -0.11, -0.00 | -0.08, 0.03 |
|  | Duty factor (% jump stride) | TrH | MD | -3.8 | -9.0 | -5.2 |
|  |  |  | P value | 0.56 | **0.02** | 0.19 |
|  |  |  | 95% CI | -11.4, 3.8 | -16.9, -1.2 | -12.3, 1.9 |
|  |  | LdH | MD | -2.8 | -8.2 | -5.4 |
|  |  |  | P value | 0.77 | **0.02** | 0.09 |
|  |  |  | 95% CI | -9.2, 3.7 | -15.1, -1.3 | -11.4, 0.6 |
|  | Z_CM_ (m) | TrH | MD | 0.10 | 0.20 | 0.10 |
|  |  |  | P value | **0.01** | **0.00** | **0.01** |
|  |  |  | 95% CI | 0.03, 0.18 | 0.12, 0.29 | 0.03, 0.17 |
|  |  | LdH | MD | 0.10 | 0.21 | 0.10 |
|  |  |  | P value | **0.00** | **0.00** | **0.00** |
|  |  |  | 95% CI | 0.04, 0.17 | 0.13, 0.28 | 0.03, 0.17 |
|  | $\dot{Z}_{CM}$ time (% jump stride) | TrH | MD | -3.1 | -6.0 | -2.9 |
|  |  |  | P value | 0.38 | **0.03** | 0.40 |
|  |  |  | 95% CI | -8.5, 2.2 | -11.6, -0.5 | -7.9, 2.1 |
|  | $\dot{Z}_{CM}$ (m/s) | TrH | MD | 0.38 | 0.76 | 0.37 |
|  |  |  | P value | 0.12 | **0.00** | 0.10 |
|  |  |  | 95% CI | -0.08, 0.85 | 0.26, 1.26 | -0.06, 0.81 |
|  |  | LdH | MD | 0.33 | 0.77 | 0.44 |
|  |  |  | P value | 0.14 | **0.00** | **0.03** |
|  |  |  | 95% CI | -0.08, 0.74 | 0.32, 1.21 | 0.03, 0.85 |
|  | $\ddot{Z}_{CM}$ time (% jump stride) | TrH | MD | -3.1 | -6.7 | -3.7 |
|  |  |  | P value | 0.29 | **0.01** | 0.12 |
|  |  |  | 95% CI | -7.8, 1.7 | -11.7, -1.8 | -8.1, 0.8 |
|  | A1 stride vel (m/s) | TrH | MD | 0.35 | 1.00 | 0.65 |
|  |  |  | P value | 0.87 | **0.04** | 0.19 |
|  |  |  | 95% CI | -0.55, 1.25 | 0.05, 1.96 | -0.25, 1.56 |
|  | Jump stride vel (m/s) | TrH | MD | 0.35 | 1.01 | 0.65 |
|  |  |  | P value | 1.00 | 0.05 | 0.21 |
|  |  |  | 95% CI | -0.62, 1.33 | -0.01, 2.02 | -0.26, 1.57 |
|  | FL A1 stance duration (s) | TrF | MD | -0.01 | -0.05 | -0.03 |
|  |  |  | P value | 0.92 | **0.01** | **0.04** |
|  |  |  | 95% CI | -0.05, 0.02 | -0.08, -0.01 | -0.06, 0.00 |
|  |  | LdF | MD | -0.02 | -0.04 | -0.03 |
|  |  |  | P value | 0.63 | **0.02** | 0.13 |
|  |  |  | 95% CI | -0.05, 0.02 | -0.08, -0.01 | -0.06, 0.01 |
| Engaged | Max hock flex take-off time (% jump stride) | TrH | MD | -2.3 | -4.2 | -2.0 |
|  |  |  | P value | 0.39 | **0.04** | 0.48 |
|  |  |  | 95% CI | -6.2, 1.6 | -8.3, -0.2 | -5.6, 1.7 |
|  | Max stifle flex take-off time (% jump stride) | TrH | MD | -2.9 | -5.2 | -2.3 |
|  |  |  | P value | 0.20 | **0.01** | 0.35 |
|  |  |  | 95% CI | -6.9, 1.1 | -9.4, -1.0 | -6.1, 1.5 |
|  |  | LdH | MD | -3.0 | -4.5 | -1.5 |
|  |  |  | P value | 0.15 | **0.03** | 0.74 |
|  |  |  | 95% CI | -6.7, 0.8 | -8.5, -0.5 | -5.0, 1.9 |
|  | Max HL shortening take-off time (% jump stride) | TrH | MD | -3.0 | -5.0 | -2.0 |
|  |  |  | P value | 0.12 | **0.01** | 0.38 |
|  |  |  | 95% CI | -6.6, 0.6 | -8.7, -1.3 | -5.4, 1.4 |


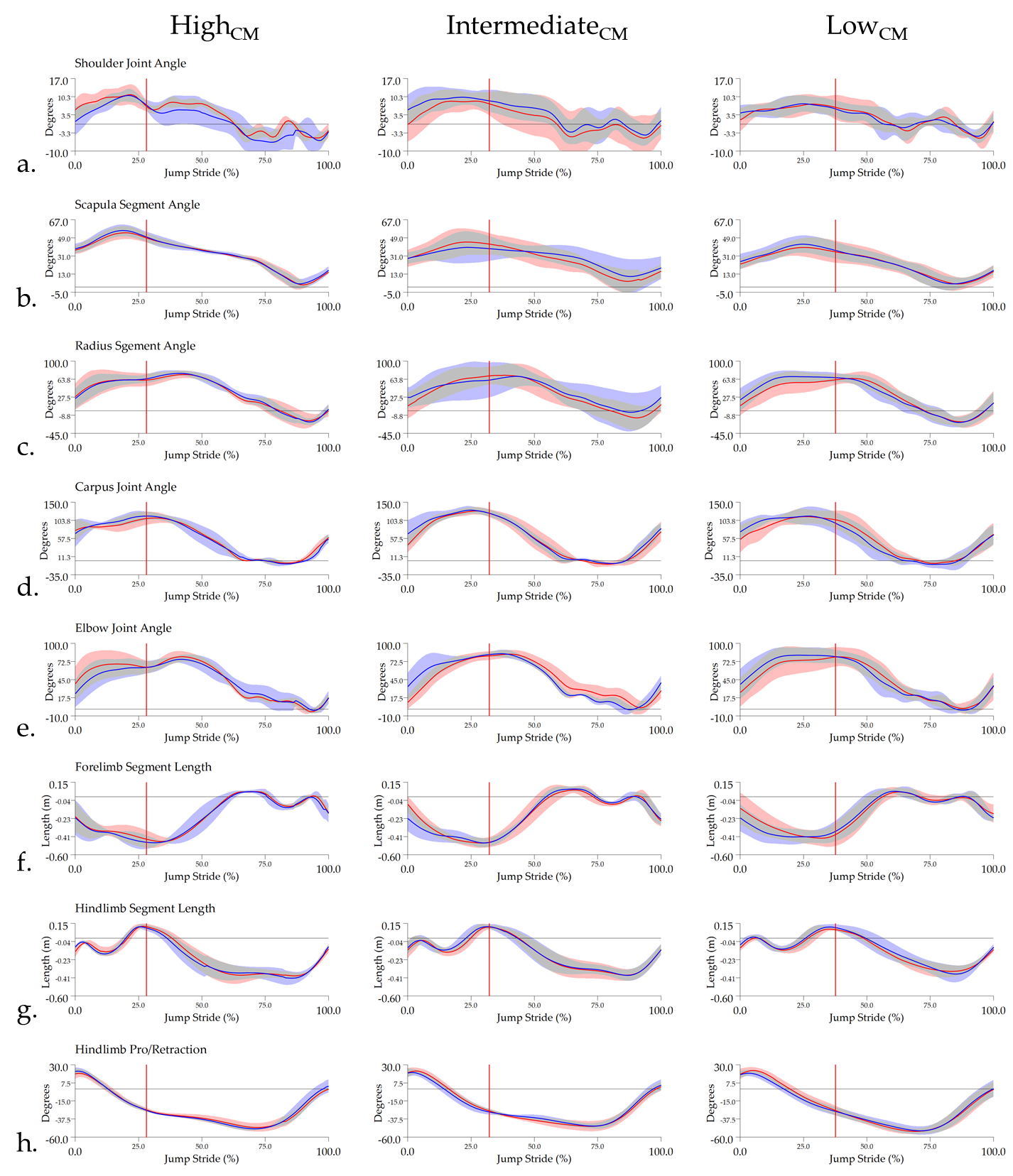


**Figure S1**: Mean and standard deviation time angle curves (˚) for kinematic variables within the joint articulation theme. a) shoulder joint b) scapula segment c) radius segment d) carpus joint e) elbow joint and h) HL segment pro/retraction angles. Mean and standard deviation data for f) FL and g) HL segment lengths (m). Data are presented in separate columns for High_CM_, Intermediate_CM_, and Low_CM_ groups and are normalised to jump stride duration. Red vertical lines represent the average HL lift-off event within each group. Mean data are presented for LdH (red line) and TrH (blue line), with shaded areas representing the standard deviation for each limb.


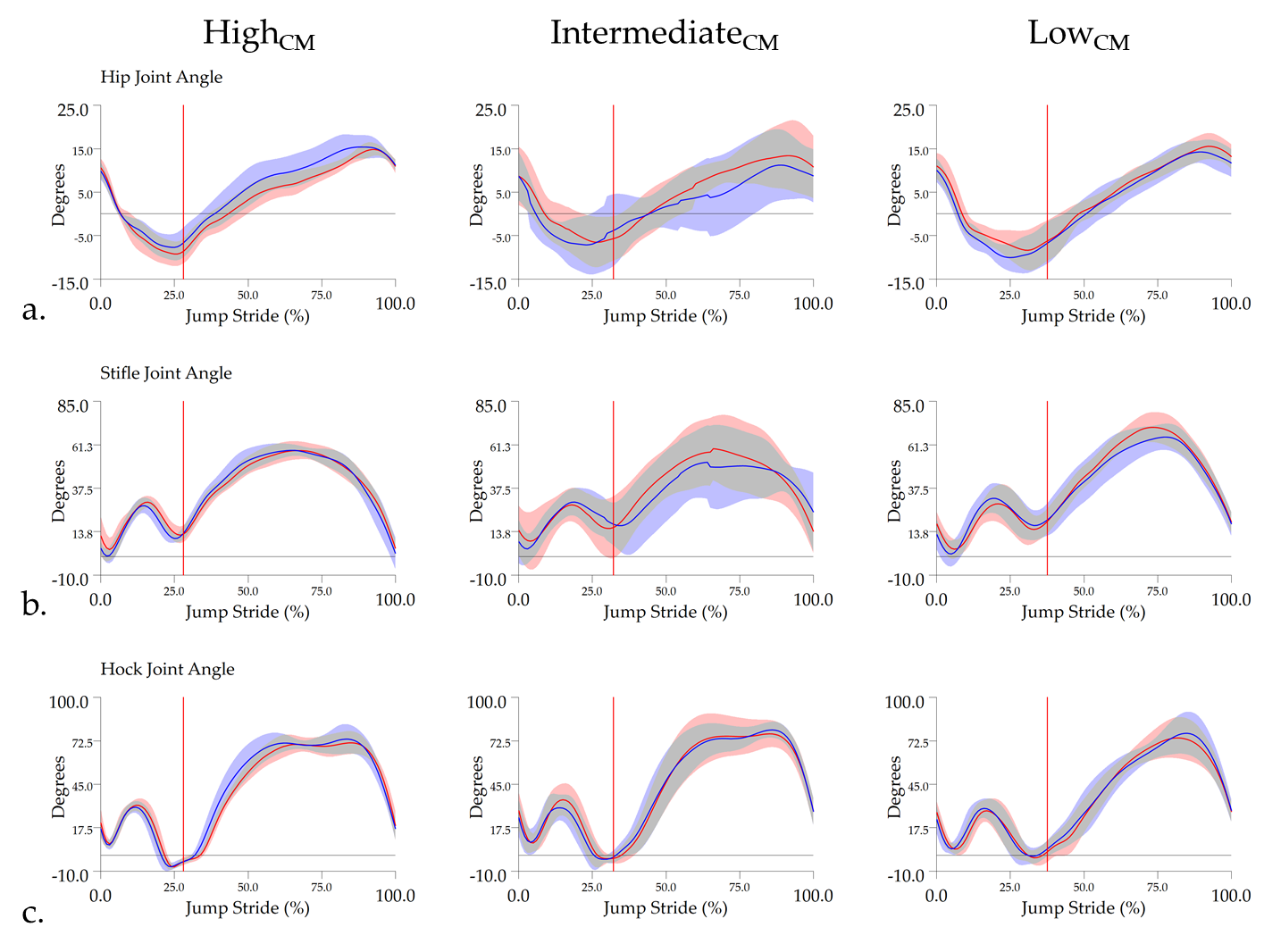


**Figure S2**: Mean and standard deviation time angle curves (˚) for kinematic variables within the engagement theme. a) hip b) stifle c) hock joints. Data are presented in separate columns for High_CM_, Intermediate_CM_, and Low_CM_ groups and are normalised to jump stride duration. Red vertical lines represent the average HL lift-off event within each group. Mean data are presented for LdH (red line) and TrH (blue line), with shaded areas representing the standard deviation for each limb.


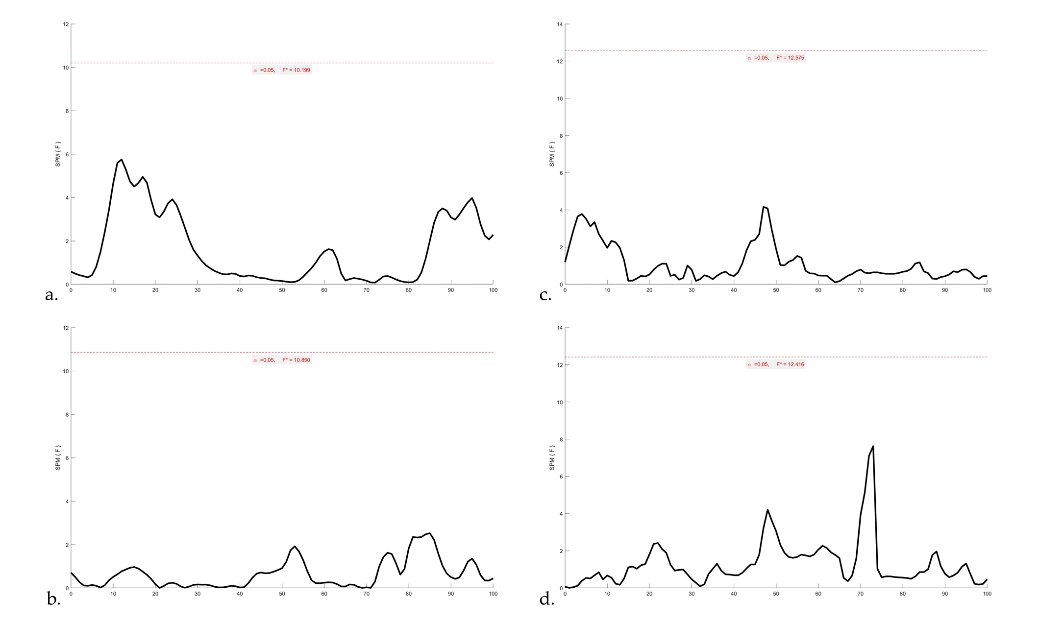


**Figure S3:** SPM results for gluteal sEMG waveforms from a) LdH A1 stride b) TrH A1 stride c) LdH jump stride d) TrH jump stride.


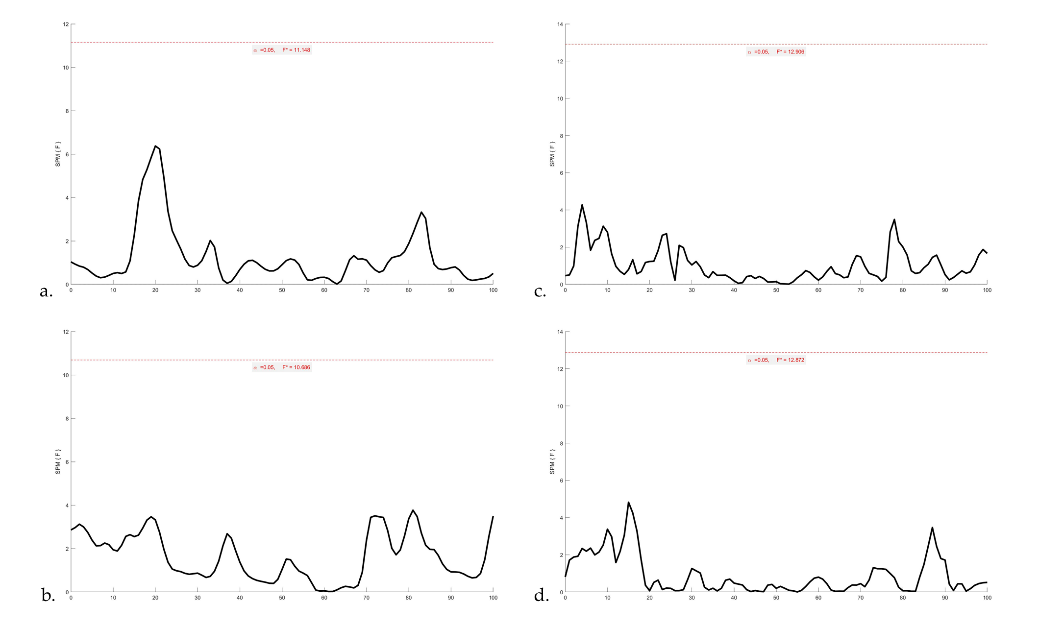


**Figure S4:** SPM results for biceps femoris sEMG waveforms from a) LdH A1 stride b) TrH A1 stride c) LdH jump stride d) TrH jump stride.


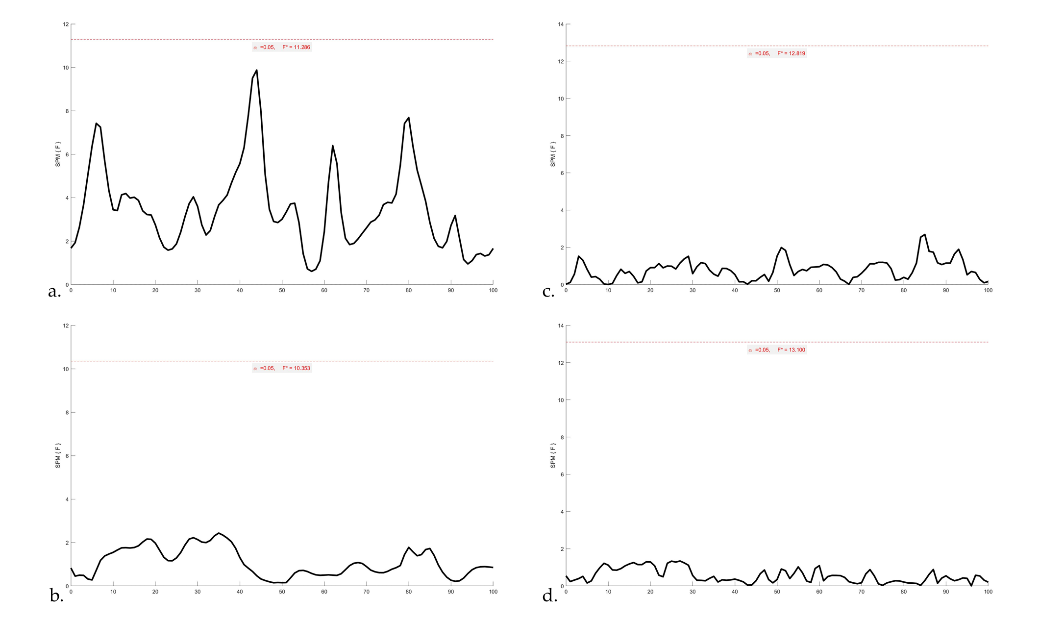


**Figure S5:** SPM results for triceps sEMG waveforms from a) LdH A1 stride b) TrH A1 stride c) LdH jump stride d) TrH jump stride.

**Table S3**. Pairwise comparisons for sEMG activity timing variables where a significant main effect was found between groups. Between group differences are presented for each variable as mean difference (MD), P-values and 95% confidence intervals (95% CI). Significant differences between groups are denoted by bold text.

| Muscle | Stride | sEMG Variable (% stride) | Limb |  | High_CM_ – Int._CM_ | High_CM_ – Low_CM_ | Int_CM_. – Low_CM_ |
| --- | --- | --- | --- | --- | --- | --- | --- |
| Gluteal | A1 | A1 activity offset | TrH | MD | -22.4 | -26.4 | -4.0 |
|  |  |  |  | P value | **0.03** | **0.04** | 1.00 |
|  |  |  |  | 95% CI | -42.8, -2.1 | -51.3 -1.5 | -28.9, 20.9 |
|  | Jump | Activity duration | TrH | MD | -15.1 | -30.5 | -15.4 |
|  |  |  |  | P value | 0.41 | **0.03** | 0.39 |
|  |  |  |  | 95% CI | -42.2, 12.0 | -57.6, -3.4 | -42.5, 11.7 |
|  |  |  | LdH | MD | -15.3 | -39.3 | -24.0 |
|  |  |  |  | P value | 0.42 | **0.01** | 0.09 |
|  |  |  |  | 95% CI | -42.6, 12.0 | -68.1, -10.5 | -51.4, 3.3 |
|  |  | Landing activity onset | TrH | MD | 8.8 | 14.6 | 5.8 |
|  |  |  |  | P value | 0.20 | **0.02** | 0.50 |
|  |  |  |  | 95% CI | -3.7, 21.3 | 2.1, 27.1 | -5.7, 17.4 |
|  |  |  | LdH | MD | 1.6 | 11.8 | 10.2 |
|  |  |  |  | P value | 1.0 | **0.05** | 0.05 |
|  |  |  |  | 95% CI | -9.7, 12.9 | 0.0, 23.6 | -0.1, 20.6 |
| Biiceps femoris | A1 | A1 activity offset | TrH | MD | -17.9 | -0.6 | 17.3 |
|  |  |  |  | P value | **0.04** | 1.00 | 0.10 |
|  |  |  |  | 95% CI | -34.5, -1.4 | -20.9, 19.7 | -3.0, 37.6 |

**Table S4**: Correlations between kinematic variables were significant between group differences were observed and the discriminative performance indicator, Z_CM_. Pearson correlation coefficients (rho) are presented for each comparison.

| **Theme** | **Kinematic outcome measure** |  | **Z_CM_ (m)** | |
| --- | --- | --- | --- | --- |
|  |  | **Limb** | TrH/TrF | LdH/LdF |
| Joint articulation | Max scapula angle time (% jump stride) | TrF | **-.72^**^** | **-.68^**^** |
|  |  | LdF | -0.40 | -0.43 |
|  | Max radius angle (°) | TrF | -0.18 | -0.22 |
|  |  | LdF | -0.02 | -0.03 |
|  | Max HL retraction (°) | TrH | 0.46 | 0.42 |
|  |  | LdH | 0.48 | .**53**^*^ |
|  | Max HL shortening time (% jump stride) | TrH | -0.31 | -0.34 |
|  |  | LdH | -0.40 | -.**52**^*^ |
| Impulsion | $\dot{Z}_{CM}$ (m/s) | TrH | **.92^**^** | **.90^**^** |
|  |  | LdH | **.88^**^** | **.93^**^** |
|  | $\dot{Z}_{CM}$ time (% jump stride) | TrH | **-.63^*^** | **-.57^*^** |
|  |  | LdH | -0.36 | -0.30 |
|  | $\ddot{Z}_{CM}$ time (% jump stride) | TrH | **-.77^**^** | **-.75^**^** |
|  |  | LdH | -0.50 | -0.38 |
|  | HL A1 stance duration (s) | TrH | **-.62^*^** | **-.65^*^** |
|  |  | LdH | **-.61^*^** | **-.67^**^** |
|  | A1 stride vel (m/s) | TrH | **-.61^*^** | **-.65^*^** |
|  |  | LdH | -0.47 | -.64^**^ |
|  | Duty factor (% jump stride) | TrH | **-.65^**^** | **-.67^**^** |
|  |  | LdH | **-.59^*^** | **-.66^**^** |
|  | FL A1 stance duration (s) | TrF | **-.62^*^** | **-.67^**^** |
|  |  | LdF | **-.58^*^** | **-.67^**^** |
| Engagement | Max hock flex take-off time (% jump stride) | TrH | **-.79^**^** | **-.68^**^** |
|  |  | LdH | **-.55^*^** | **-.54^*^** |
|  | Max stifle flex take-off time (% jump stride) | TrH | **-.83^**^** | **-.79^**^** |
|  |  | LdH | **-.61^*^** | **-.64^**^** |
|  | Max HL shortening take-off time (% jump stride) | TrH | **-.78^**^** | **-.75^**^** |
|  |  | LdH | **-.62^*^** | **-.58^*^** |
