# Supplementary figures and images for: Muscle Function and Kinematics during Submaximal Equine Jumping: What Can Objective Outcomes Tell Us about Athletic Performance Indicators?
Source: Animals (Basel). 2021 Feb 5;11(2):414. doi: 10.3390/ani11020414 (PMC7915507; doi:10.3390/ani11020414)

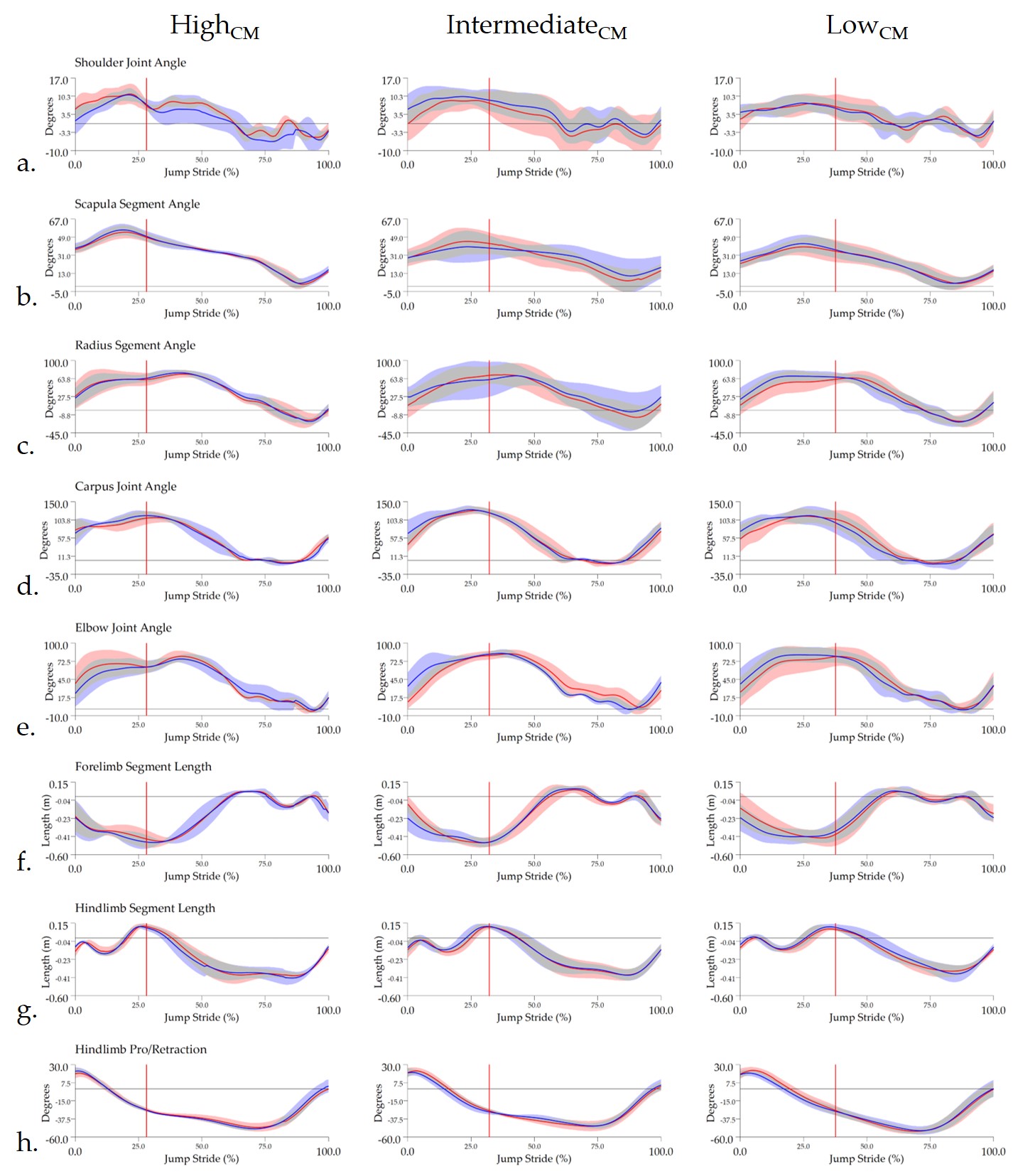

Supplement: Supplementary file 1 [file animals-11-00414-s001.zip › St George et al Supplementary Info/Supplementary Figure S1.jpg]

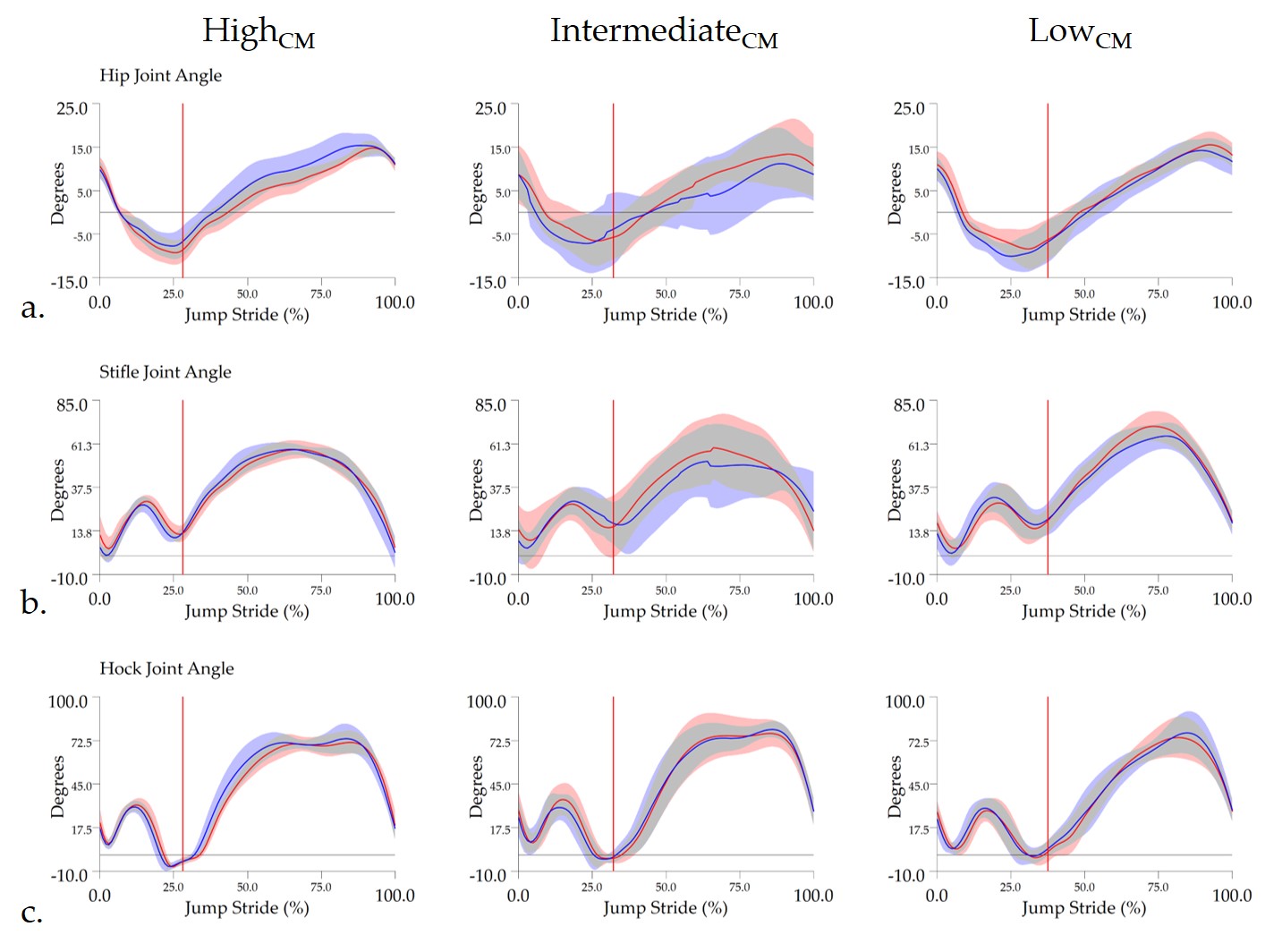

Supplement: Supplementary file 1 [file animals-11-00414-s001.zip › St George et al Supplementary Info/Supplementary Figure S2.jpg]

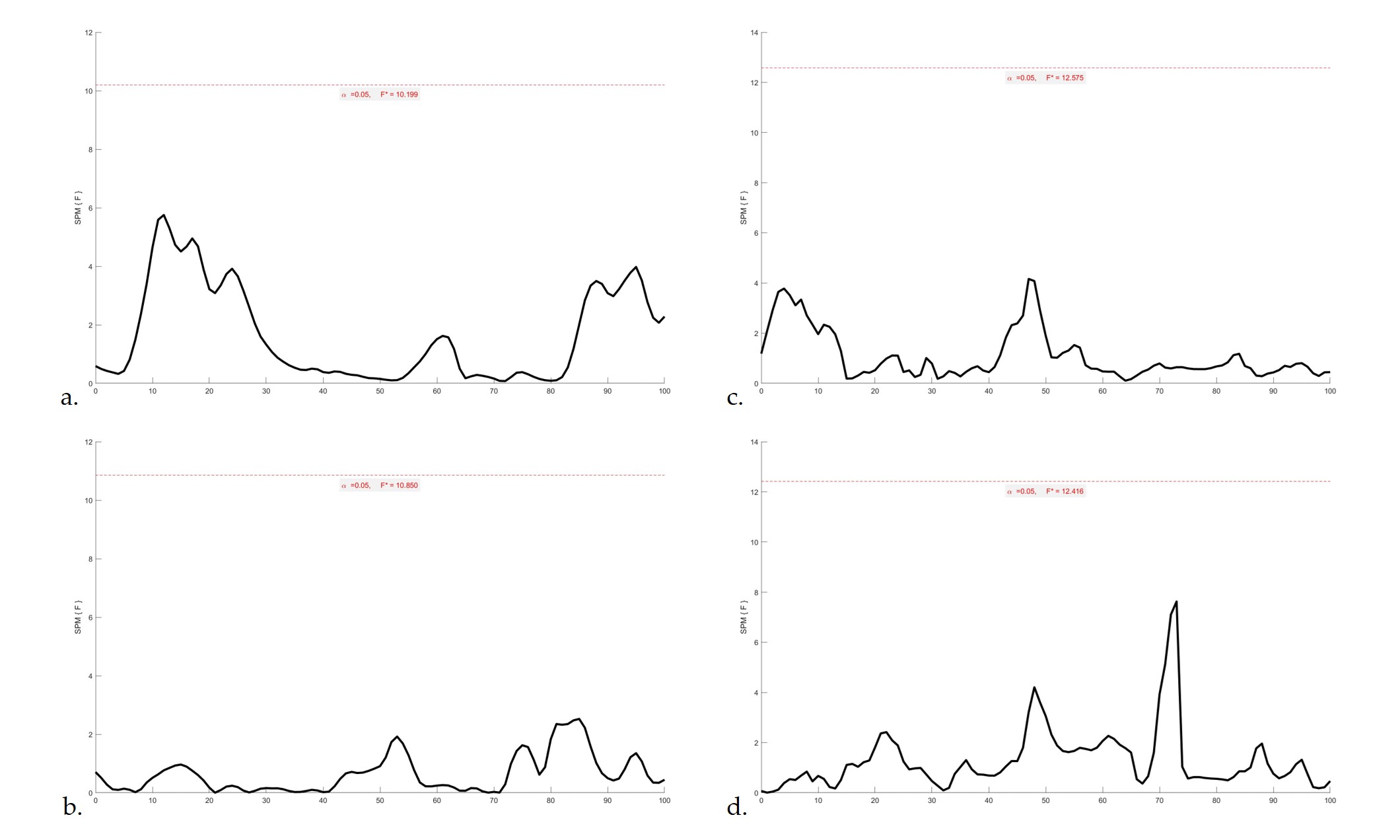

Supplement: Supplementary file 1 [file animals-11-00414-s001.zip › St George et al Supplementary Info/Supplementary Figure S3.jpg]

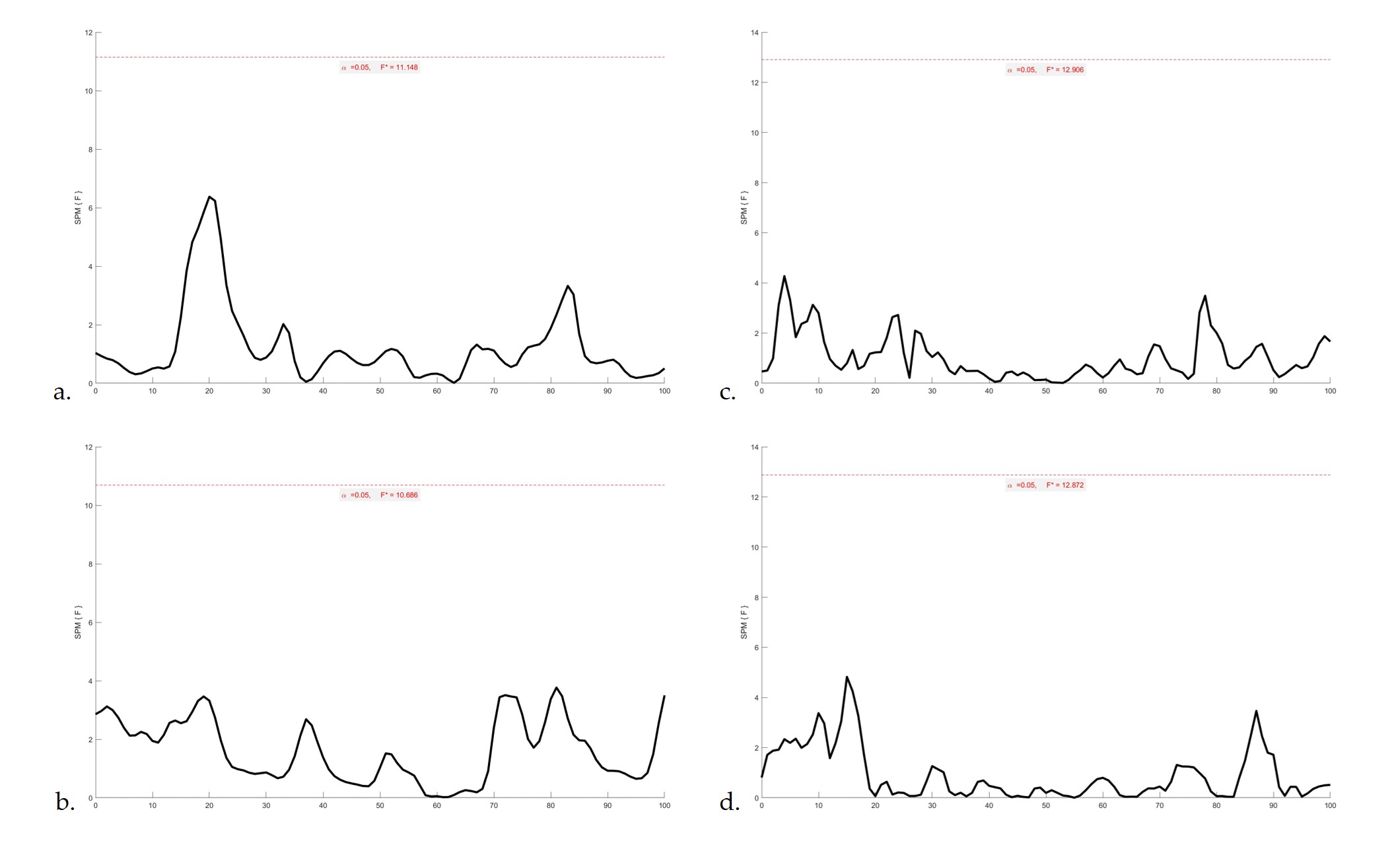

Supplement: Supplementary file 1 [file animals-11-00414-s001.zip › St George et al Supplementary Info/Supplementary Figure S4.jpg]

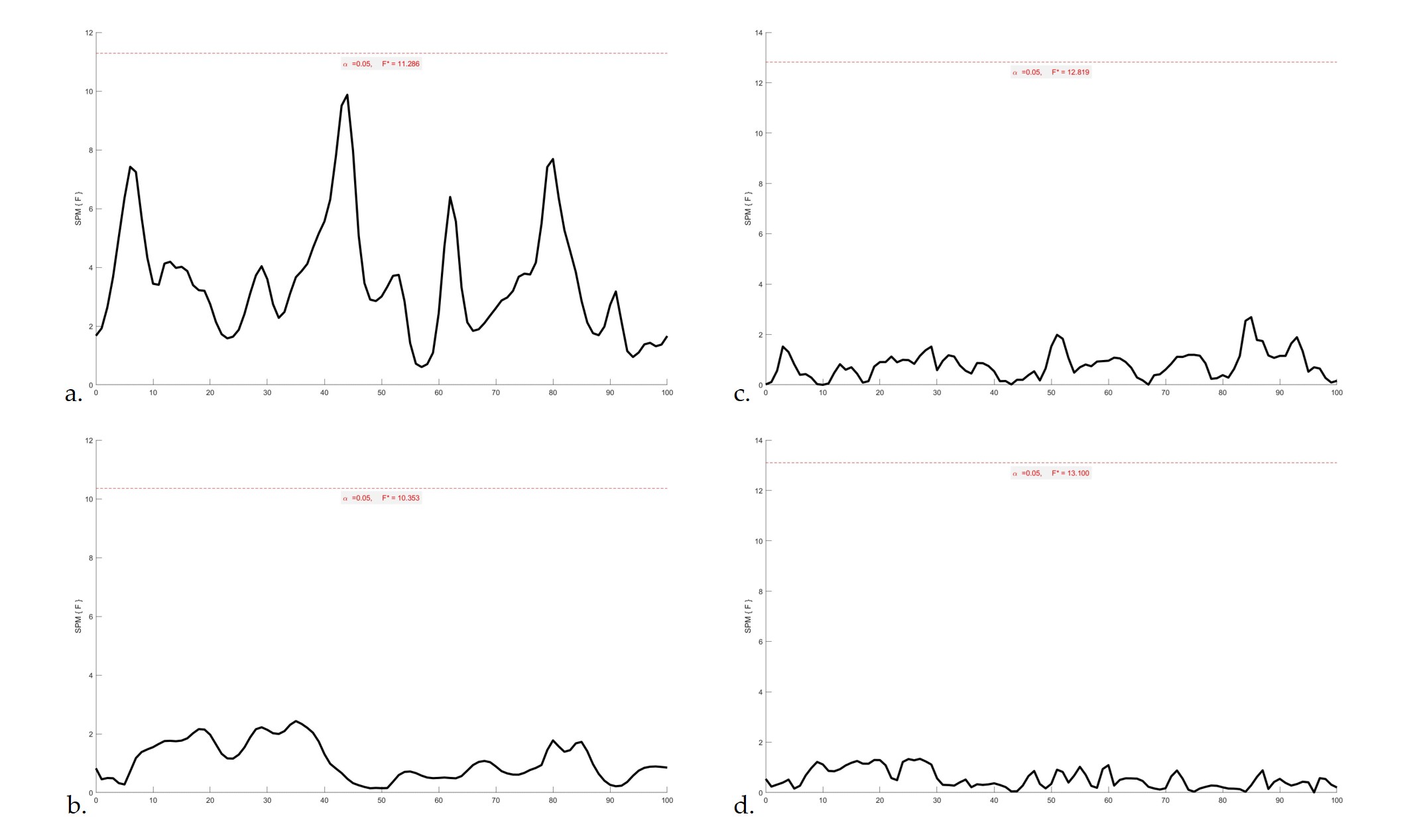

Supplement: Supplementary file 1 [file animals-11-00414-s001.zip › St George et al Supplementary Info/Supplementary Figure S5.jpg]
